# Probing the role of cell wall feruloylation during maize development by differential expression of an apoplast targeted fungal ferulic acid esterase

**DOI:** 10.1371/journal.pone.0240369

**Published:** 2020-10-09

**Authors:** Marcia M. de O. Buanafina, M. Fernanda Buanafina, Sue Dalton, Phillip Morris, Marissa Kowalski, Manav K. Yadav, Lindsay Capper

**Affiliations:** 1 Department of Biology, The Pennsylvania State University, University Park, PA, United States of America; 2 Institute of Grassland and Environmental Research, Aberystwyth, United Kingdom; University of Sao Paulo, BRAZIL

## Abstract

While many aspects of the growth of maize are well understood, the role of cell wall feruloylation particularly during internode elongation has not been firmly established, but results so far indicate that it has significant implications for both biofuel feedstock conversion and for crop yield. The growth of the cell wall is achieved by synthesis, integration and cross-linking between wall polymers. As ferulate oxidative coupling of arabinoxylan side chains constitutes a significant type of cross-link in grass cell walls, it is expected to have a crucial role in plant growth. Making use of plants expressing an apoplast targeted *Aspergillus niger* FAEA under the control of either a constitutive or an inducible promoter, the role of cell wall feruloylation in maize internode expansion was investigated. Analysis of FAEA expressing plants showed that where FAEA was targeted to the apoplast under a constitutive promoter, plants varied in stature either from semi-dwarf plants with a 40–60% height reduction, to extreme dwarf mutants with over 90% reduction in plant heights compared to controls. Results indicate that disruption of cell wall feruloylation by FAEA occurs before the start of rapid internode expansion is initiated and affects the normal course of internode elongation, resulting in short internodes and dwarfed plants. In contrast, when under the inducible *Lm See1* senescence promoter, FAEA activity was found to be low up to the VT stage of development but increased significantly at the VR stage as plants began to senesce, strongly suggesting that normal cell wall feruloylation is required for the process of internode expansion. In addition, with apoplast targeted expression of FAEA under control of the senescence enhanced promoter it was possible to demonstrate decreased cell wall feruloylation without affecting internode expansion or other aspects of plant development.

## Introduction

The growth of plant cells can be defined as the irreversible increase in cell volume that is brought about by water uptake and controlled by the mechanical properties of the cell wall including wall-loosening followed by wall-stiffening [1]. These processes are achieved by the cell wall regulating among other things, its polysaccharide composition and levels of cross-linking [2] which are responsible for maintaining the strength of the expanding wall to withstand the forces that induce their growth.

Some specific features of cell walls in maize, as in other Poaceaes include the high level of arabinoxylans (~ 30%) and relatively high levels of hydroxycinnamates (HCAs) (0.5–1.5% dry weight), consisting mainly of ferulic and *p*-coumaric acids which are not restricted to lignified cells but are also present, in significant amounts, in the cell walls of growing tissues [3]. Arabinoxylans consist of a (1–4)-liked β-ᴅ-xylanopyranosyl backbone substituted with α-ʟ-arabinofuranozyl at the xylopyranosyl *O*-3 and *O*-2 positions, as the major substituents. Other additional substitutions include α-ᴅ-glucuronic acid or their 4-O-methyl ester groups which have been particularly observed in maize and sorghum arabinoxylan (AX) [4–6]. Ferulic acid (FA) acylates a portion of arabinose residues via an ester-linkage at the C5-hydroxyl group of α-ʟ-arabinozyl residues of arabinoxylan and some ferulates are also ether- or C-C linked to lignin [7–9]. Upon oxidative coupling, mediated by peroxidase/H_2_O_2_, over 50% of cell wall esterified ferulates form different dimers [10–12] and trimers [13, 14] cross-linking AX by creating inter and intra-molecular bonds between previously separated AX chains. Ferulate coupling with lignin also leads to cross-linking polysaccharides and lignin [8]. Studies support the proposition that dimerization of ferulic acids take place within the cell wall as well as intracellularly [15–17].

In maize, internodal growth is basipetal with the intercalary meristem localized at the base of the internode which remains active until the very end of internode elongation [18, 19]. According to Scobbie et al. (1993), Kende et al. (1998) and Zhang et al. (2014) [18, 20, 21] elongating internodes have four distinct regions: the basal region (containing the intercalary meristem), an elongation zone (where primary cell walls are deposited), a transition zone (where secondary synthesis begins) and at the upper end the maturation zone (where secondary cell wall synthesis predominates). It is quite a complex process and of relevant importance as it determines, to a significant extent, not only the ability of the plant to compete for light (which determines the distribution of leaves) but also the relationship between vegetative and reproductive growth. The process of internode cell elongation in maize does not occur before the reproductive phase has started [22] and involves the correct orientation of newly synthesized cellulosic microfibrils transverse to the cell elongation axis and the deposition of secondary cell wall constituents (such as glucose, xylose, lignin) which accumulate rapidly shortly before internode elongation ends. Ester and ether-linked ferulates are deposited into both primary and secondary walls. Esters start to be deposited before ether-linked ferulates and reach around 60% of their deposition by the time secondary cell wall formation begins [21, 23, 24].

Since the first reports on the presence of ferulates in plant cell walls, much work has revolved around establishing their roles within cells. Because the ferulates that cross-link polysaccharides alter the physicochemical properties of the cell wall building blocks in which they occur, it has been suggested that they will control the rate of wall extension and therefore growth rate [25, 26]. Feruloylation has also been suggested to make the wall more resistant to microbial digestion, hindering cell wall degradability [27, 28], and ultimately playing a role in plant protection against pests and pathogens [29, 30], and to be involved in cell wall assembly [9], and promotion of tissue cohesion by mediating linkage between the middle lamella and primary cell wall [31, 32]. Ferulate esters have also been held responsible for being the initiation site for lignin deposition in grasses [33]. Although bringing new insights regarding the role of feruloylation in these various processes, these studies have been in most cases based on correlation between two processes and not by alteration of cell wall feruloylation.

Buanafina et al. (2010) [34] showed that the level of cell wall ferulates monomers and dimers can be altered *in planta* by targeted expression of an *Aspergillus niger* ferulic acid esterase (FAEA) to the apoplast, which targets inter-AX chain interactions as cell wall polymers are being formed. Feruloyl esterase represent quite a diverse group of carboxylic acid esterases that catalyze the cleavage of 1->5 ester bond between arabinose and ferulic acid, releasing ferulate monomers and dimers from cell walls [35]. Using this strategy of directly manipulating the level of cell wall feruloylation by targeted expression of FAEA it has been possible to test the role of feruloylation on plant processes such as cell wall degradability [34, 36, 37], plant insect resistance [38], and plant growth and cell wall turnover [39].

The aim of this study was to investigate the role of feruloylation in internode development and leaf morphology in maize. This was accomplished using transgenic plants expressing the *A*. *niger* FAEA gene under the control of either a constitutive actin (*p*IGB6) or inducible *LmSee1* (*p*JQ5) promoter, targeted to the apoplast, to reduce feruloylation levels *in muro*. The results show that disruption of feruloylation before internode rapid expansion is initiated by FAEA expression, restrain internode expansion and results in dwarfed plants suggesting that normal cell wall feruloylation is required for the internodes to expand.

## Materials and methods

### Vector construction and maize transformation

The three vector constructs used in this study carried a genomic clone of the *Aspergillus niger faeA* gene as described previously [34, 40]. In *p*IGB6 and *p*INHΔ, *fae*A was under the rice actin promoter (Act1::*fae*A) and in *p*JQ5 under the *Lolium multiflorum* senescence inducible promoter (*Lm*::*fae*A), with FAEA targeted to the apoplast in *p*IGB6 and *p*JQ5 or to the vacuole in *p*INHΔ. Evidence for the successful targeting of FAEA to the vacuole with an identical aleurain/NPIR vacuole targeting vector, but a slightly different actin promotor has been demonstrated previously in *Festuca* [37], see also Buanafina et al. (2015) [40] for the detailed structure of *p*INHΔ.

The FAEA constructs were introduced into immature embryo cultures of maize hybrid genotype Hi-II (A188 x B73) using protocols from Iowa State University [41] by co-bombardment with *p*UBA carrying a maize ubiquitin gene promoter-*bar* chimeric gene [42]. T0 plants were regenerated from bialophos resistant calli and screened by PCR for the presence of the FAEA gene and for FAEA enzyme activity. PCR and FAEA positive plants were back crossed to B73 or to negative FAEA lines to generate T1 TQ (*p*JQ5) and TG (*p*IGB6) seeds or TΔ (*p*INHΔ) T_0_ plants. Transgenic and control plants were analyzed at the V6 (six expanded leaf stage—at this stage of development, the growing point and tassel are above the soil surface and the stalk is beginning to elongate), and at the V9 (nine expanded leaves), V15 (fifteen expanded leaves), VT (tasseling) and VR (reproductive) developmental stages, according to Iowa State University “How a corn plant develops” special report [43]. Plants at the VT stage were harvested 5–6 days after the start of pollen shed, and at VR around 6 weeks after pollination (at the R5 denting stage). For transgenic and control lines, all internodes were sampled at each specific stage.

### Plant material and growth conditions

Reported in this work are 18 TG plants constitutively expressing apoplastic targeted FAEA, 9 TQ plants expressing senescence inducible apoplastic targeted FAEA and 4 TΔ plants constitutively expressing vacuole targeted FAEA. Of the TG plants 5 were extreme dwarf mutants, (>90% reduction in plant height) of which 3 were studied in some detail separately, 2 plants were slight dwarf mutants (<10% reduction in plant height) which were not studied further, and 11 plants were intermediate dwarf mutants (40–60% reduction in plant height). Of these 11 TG and 8 TQ plants, TG 1, 4 and 10 and TQ 4, 6 and 11 were sacrificed to determine developmental FAEA activities and HCA levels at different stages of plant development. Plants TG 5,7,8,9 and TQ 8, 9,12 and 13 were used as replicates for determining plant growth but TQ12 was grown further to the R5 stage before harvesting and used in determining FAEA activities, and changes in internode cell morphology at this stage of development. The remaining 4 semi and extreme dwarf TG mutants were examined for changes in internode anatomy, the level of FAEA activities in different sections of internodes and cellular morphology. Plants TG6 and TG9 and TQ10 and TQ13 were analyzed in detail for plant growth, stem morphology, leaf, internode and epidermal morphology, FAEA activity in leaves and internodes, ester and total HCAs and cell wall sugars in internodes at the VT stage of development. Plants TQ15 and TQ16 were used to determine the level of FAEA activities in different sections of internodes. Plants TΔ 1–4 were examined for levels of FAEA activities in different tissues. Because transgenic lines were grown in several batches, all FAEA expressing plants were allocated individual control plant partners that were grown, harvested stored and analyzed in parallel with the transformants. T1 control and positive FAEA plants were grown under glasshouse conditions (29°C day: 27°C night, with supplementary 1000W metal halide lamps 16h/8h light/night cycle) at the Pennsylvania State University.

### Growth measurements and tissue sampling

Plant growth rates were calculated as the mean weekly increase in plant height. Height was measured once a week until plants stopped elongating (VT stage).

For FAEA activity determination, whole leaf blades (without midribs) were chopped, mixed and samples collected. Analysis of internodes was carried out on whole longitudinal sections, to take into account cell wall compositional differences along the internodes. All the remaining material from individual leaves and internodes was used to measure ester and ester+ether linked cell wall HCA and cell wall sugars, and to determine sugar release by cellulase, and were prepared as described previously [39]. Fresh weights, leaf and internode length, leaf and internode width/diameter, from transgenic and control plants at different developmental stages, were all determined at harvest. Subsequently, harvested and sampled tissues were stored at -80°C until used for further analysis.

### Anatomical analysis

Thin transverse sections through the central vascular bundles of the base portion of internode 6 of TG, TQ and control plants were made manually using a scalpel blade. Cross-sections were stained with either toluidine blue or phloroglucinol. Following 20–30 s treatment with 0.1% (w/v) aqueous toluidine blue solution, sections were rinsed twice in distilled water. To stain and visualize lignin, sections were treated with 3% phloroglucinol (3% mixed with 12M HCl, fresh mix, in a 1:9 part ratio) for 2–3 min. Stained cross-sections were mounted on microscope slides and visualized using an Olympus IX51/IX71 microscope attached to a CCD colour XC10 digital camera. Images were captured using cellSens Entry 1.7 Software and further processed with analySIS getIT software and Adobe Photoshop, where images were only corrected with auto levels and for brightness to match the microscope images.

### Epidermal cell dimensions and number

The spatial distribution of cell lengths and widths and the number of epidermal cells per unit area were determined from replicas of abaxial epidermal peels of fully expanded leaves of control and apoplast FAEA expressing plants TG6, TG9, TG12 and TQ10, TQ13 at the VT developmental stage, sampled 1cm from the leaf tip, in rows adjacent to the leaf midrib. Samples were prepared as described in Weyers and Travis et al. (1981) [44] and stained briefly in 0.05% (w/v) toluidine. Cell dimensions were measured under an Olympus IX51 light microscope. Images were captured and analysed as described in Buanafina et al. (2017) [39]. Area (lengths and widths) of 10 cells in rows adjacent to the midrib, were recorded for each peel, and a total of ten peels from 2–3 leaves were measured and recorded per line. The number of cells was counted within 600,000 μ^2^ areas randomly selected from 10 leaf zones from 2 leaves per line.

### Determination of FAEA activity

Soluble proteins were extracted from individual leaf blades, internodes and nodes, and 100ug protein incubated with 24mM ethyl ferulate as substrate for 24h at 28°C, and FAEA activities determined by high-performance chromatography (HPLC) as described in Buanafina et al. (2017) [39]. One Unit of FAEA activity was defined as the release of 1μg ferulic acid from ethyl ferulate in 24h at 28°C. Therefore 1 Unit = 3.6 x10^-6^ μmole FA min^-1^.

### Cell wall analysis

#### Preparation of isolated cell wall (AIR)

Leaf and internodes harvested as described in the previous section, were ground in liquid nitrogen, freeze dried and milled to a fine powder using a mixer mill (Retsch MM301) before cell wall AIR was prepared as described in Buanafina et al. (2015) [40].

#### Determination of cell wall ester and ether-linked hydroxycinnamic acids

Quantitative analysis of extracted ester and total ether+ester-linked HCAs was performed on isolated cell walls (AIR) by HPLC, as described in Buanafina et al. (2015 and 2017) [39, 40]. The internal standards 2-hydroxycinnamic acid and 3-hydroxy-4-methoxycinnamic acid (100 μg) were added for ester and total ether+ester-linked HCA analysis, respectively.

#### Determination of the cell wall sugars xylose, arabinose and glucose

High Performance Anion Exchange Chromatography (HPAEC) of hydrolysed cell wall samples was used to determine the level of the monosaccharides, arabinose, xylose and glucose in isolated cell wall AIR, based on methods from Øbro et al. (2004) [45] with modifications as described in Buanafina et al. (2012) [46].

### Cellulase-mediated glucose release from internodes

Freeze-dried powdered internode material (30 ± 0.2 mg), was re-hydrated in 1.8 ml 0.1 M sodium acetate, pH 5.5 extraction buffer and incubated at room temperature for 24 h. After centrifugation, the supernatant was removed, pellets washed twice with buffer and incubated with 160 μl cellulase (*T*. *reesei*, Sigma, 63 units/ml) and 4 ul 2% sodium azide in a total volume of 2 ml of the same buffer. Samples were incubated at 37⁰C for 48 h. Reactions were terminated by placing samples at 100°C for 5 minutes. Reducing sugars were determined by the ρ-hydroxybenzoic acid hydrazide (PAHBAH) method (Lever, 1972) [47] as described in Buanafina et al. (2010) [34].

### Statistical analysis

Statistical Analysis System (SAS) (2015) [48] software version JMP Pro 14 (SAS Institute Inc., Cary, NC) was used to perform all statistical analysis. Values in the text are means ± the standard error of the means (SEM). Bars with asterisk are significantly different from controls (Student’s *t*-test α = 0.05).

## Results

### Generation of transgenic plants expressing FAEA under a constitutive or inducible promoter and plant phenotypes

The generation of maize plants which express FAEA either constitutively, or during senescence at different stages of plant development could provide a useful system to study the role of feruloylation in internode and leaf elongation and plant developmental growth. Based on this, two constructs were made to target FAEA to the apoplast under either the constitutive rice actin promoter (*p*IGB) or under the induced *Lm* senescence promoter (*p*JQ5). In addition, FAEA was also targeted to the vacuole under the rice actin promoter. The presence of the transgene was confirmed by FAEA expression. Detailed FAEA activities and morphological analysis during development (V6 through VT) was performed on plants of male and female backcrossed T1 lines, derived from 4 independent transformation events with *p*IGB6 (TG plants), 5 events with pJQ5 (TQ plants) and 4 events with *p*INH1Δ (TΔ plants). The overall percentage of progeny expressing FAEA was 18%, with a 15% male but only a 2% female rate of transmission.

**Fig 1:**
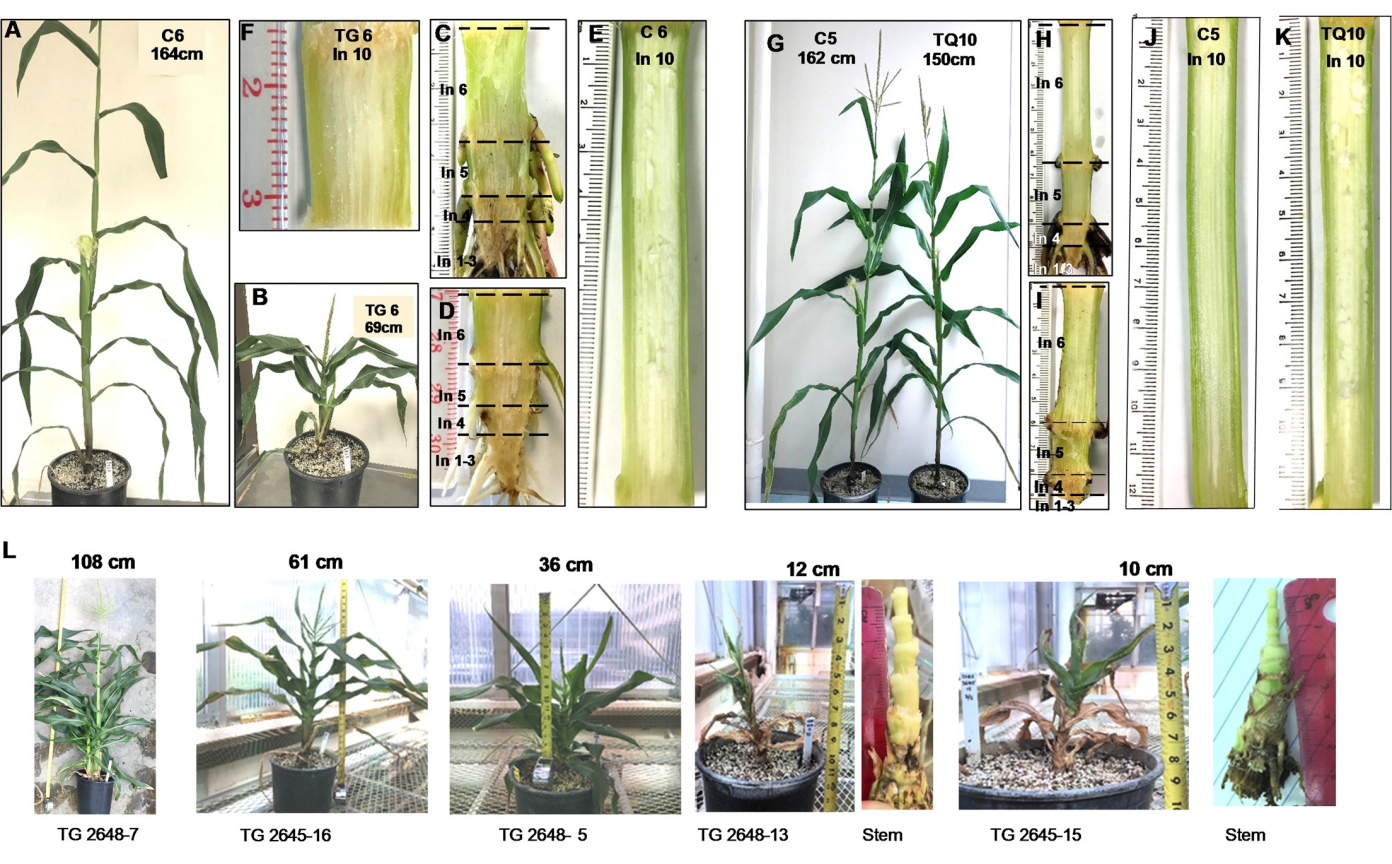
Plant morphological phenotypes of FAEA expressing TG and TQ plants at the VT stage of development. Maize plants at VT stage expressing apoplast FAEA, either constitutive (TG plants) or senescence induced (TQ plants), compared with control plants. C6 control **(A)** and TG6 stature mutant **(B)**; Internode phenotypes (**C-F)**. Internodes1-6 **(C)** and 10 **I** of C6; and Internodes1-6 **(D)** and 10 **(F)** of TG6. TQ10 plants and C5 control **(G)**. Internode phenotypes **(H-K)**. Internodes1-6 **(H)** and 10 **(J)** of C5, and Internodes1-6 **(I)** and 10 of TQ10 **(K)**. Five examples of the range of TG stature mutants generated by constitutive apoplastic FAEA expression **(L)**. All plants were grown under glasshouse conditions.

When FAEA was targeted to the apoplast under the constitutive rice actin promoter, FAEA expression started at a very early stage during plant development and before the internode fast elongation stage was initiated. All 18 TG plants were dwarfed or semi-dwarfed with statures at the VT tasselling stage ranging from 10–127 cm in height (Fig 1B–1L), in contrast to the controls I which were between 164–180 cm (Fig 1A). This resulted from shortened internode phenotypes. Examples of this are internodes 1–6 and 10 in plant TG6 (Fig 1D–1F) compared with the control plant C6 (Fig 1C–1E), at the VT stage. This trait was observed during all the developmental stages studied (V6) through to VT (S1 Fig). TG plants also produced underdeveloped tassels and ears with reduced pollen and silk production, leading to sterile plants. The dwarf phenotypes of TG lines are further illustrated in Fig 1L, and S2A–S2C, S3A Figs.

When FAEA was targeted to the apoplast under the *Lm* senescence promoter, FAEA expression was induced only after the internodes had passed the fast elongation stage, and later during leaf expansion. All 9 TQ plants examined, showed morphological phenotypes similar to the control plants, for all the parameters measured and during all the developmental stages studied (Fig 1G–1K and S1A–S1D Fig). Dwarfed phenotypes were also observed in the TΔ plants where FAEA was constitutively targeted to the vacuole (S4A Fig).

### Stem morphology phenotypes at the VT tasselling stage of development

In order to investigate whether the altered dwarfed phenotypes were associated with anatomical changes in the cell wall of tissues in the vascular bundles, cross sections through the sixth internode of TG, TQ and control plants, at the VT stage, were stained with toluidine blue and phloroglucinol and visualized microscopically. The overall vascular structure in cross sections of internode 6 of TQ plants at high magnification did not appear to be significantly different from control plants.

**Fig 2:**
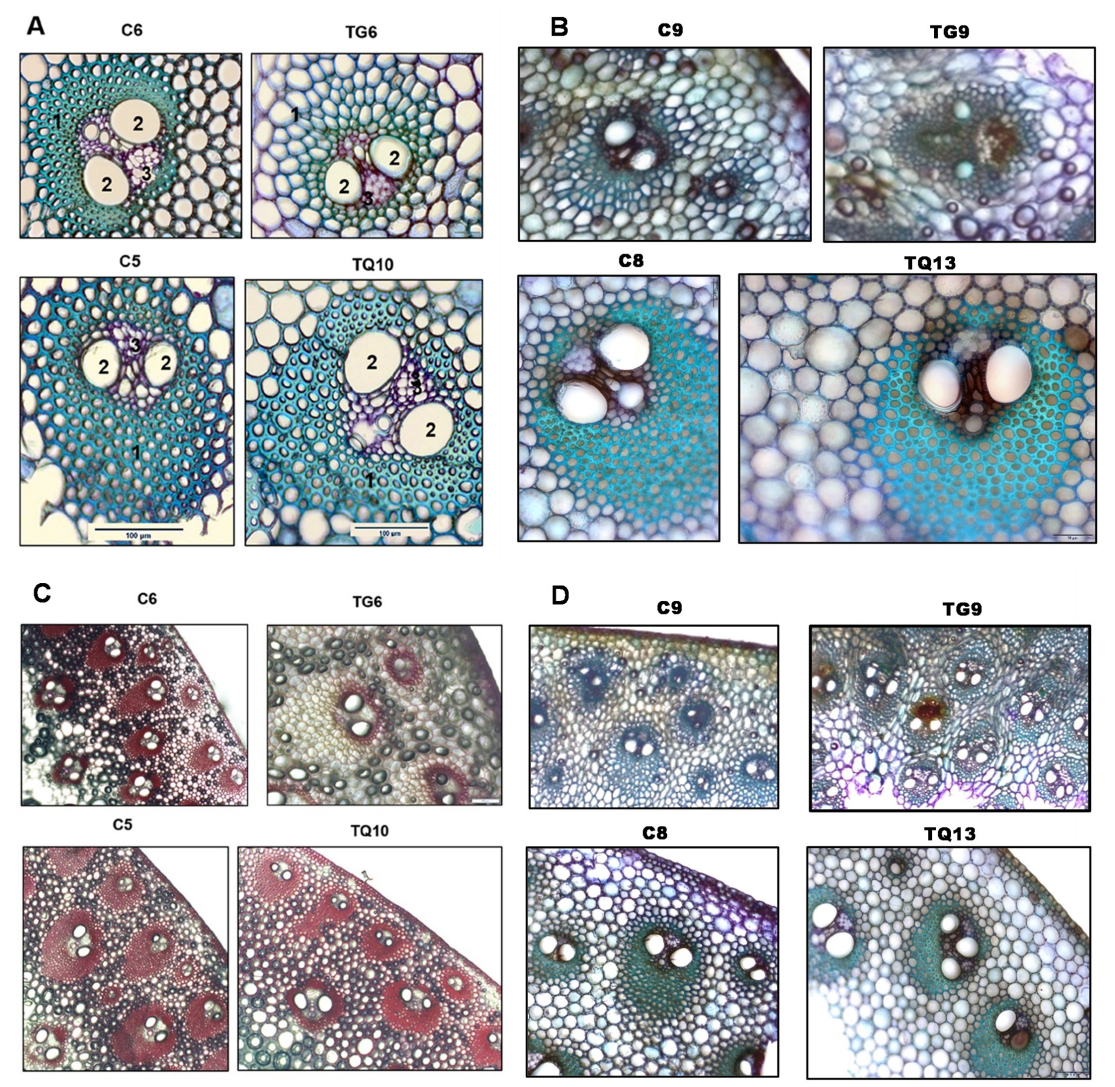
Stem morphology phenotypes of FAEA expressing TG and TQ plants at the VT tasselling stage of development. Transverse sections through the central vascular bundles of internode 6 of apoplast FAEA expressing plants TG6 and TQ10 **(A,C)**, TG9 and TQ13 **(B,D)** and corresponding control plants (C6,C9, C5,C8) at high (20X) **(A,B)** and low (4X) **(C,D)** magnification, grown under greenhouse conditions to the VT tasselling stage of development, stained with either toluidine blue **(A,B,D)** or phloroglucinol **(C).** The sclerenchyma fibres (1), xylem vessels (2) and sieve elements (3) are indicated in (A). Bars = 100 μm.

However, in the TG toluidine blue stained sections, the very highly thickened cell walls of the sclerenchyma, which is the supportive tissue between epidermis and xylem, appeared to be completely disrupted and replaced by thin walled enlarged cells similar to (but larger than), the outer parenchyma cells when compared with sections of control internodes (Fig 2A, 2B and 2D). Lower magnification phloroglucinol stained sections also showed that while the overall structure of the vascular system probably remains functionally intact in the TG transformants, it had been modified by FAEA expression and was more diffuse and less tightly structured, particularly the sclerenchyma which appeared to be almost absent in TG6 (Fig 2C). In addition, it also was observed that control and TQ internodes were more rigid and easier to section, whereas TG internodes were spongy, less rigid and fell apart during sectioning, which may reflect changes in cell-to-cell adhesion in the TG plants.

These anatomical changes in vascular bundle cell walls in internodes were found in all the extreme TG stature mutants, and to a lesser extent in sections of the semi-dwarf plants, decreasing in severity with plant height (S2A–S2C, S3B, S5A, S5B and S6 Figs). Such differences were not observed in sections of TQ internodes of plants at the VT stage, stained with toluidine blue (S5C, S5D and S6 Figs). With phloroglucinol, the TQ internodes stained with a similar intensity to the controls and were darker than the TG internodes (Fig 2C and S5E–S5H Fig), indicating that the TG vascular bundles at the VT stage contain reduced coniferaldehyde end-groups compared with control and TQ plants.

### Growth and developmental phenotype of FAEA expressing plants

**Fig 3:**
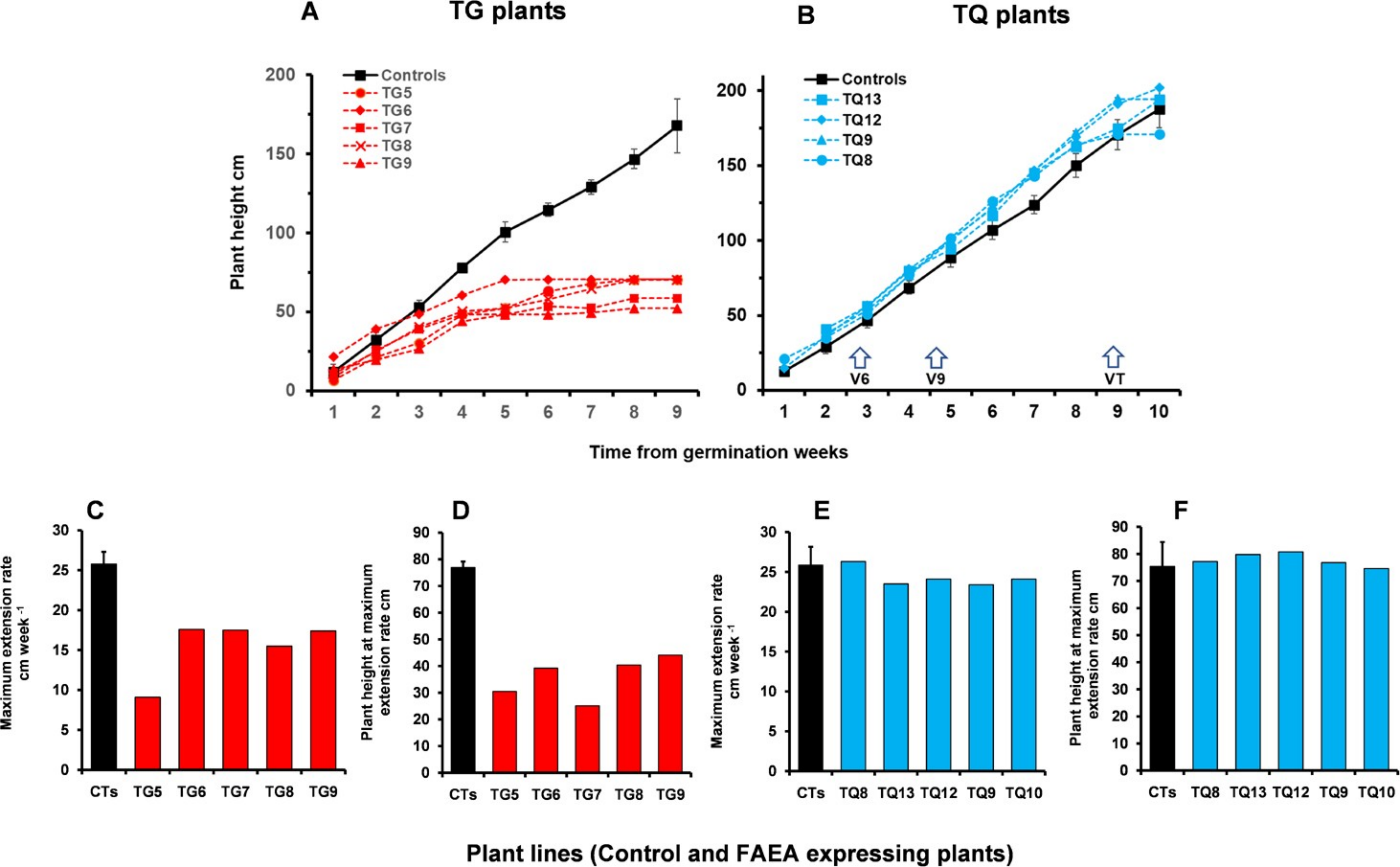
Growth of FAEA expressing TG and TQ plants. Comparison of the growth of individual maize plants, measured as the increase in plant height **(A,B)**, expressing apoplast FAEA, either constitutive in TG plants **(A)** or senescence induced in TQ plants **(B)**, and indicating the age of plants at three developmental stages (V6 to VT). Maximum weekly extension rate, of TG **(C)** and TQ **(E)** plants, and plant height at maximum extension rate of TG **(D)** and TQ **(F)** plants, compared with the mean growth of 2 control plants [mean ± sem (n = 4)]. Plant heights were measured weekly to the VT tasselling stage of development.

Plant growth, as measured by the increase in height over time was much lower in the semi- dwarf TG FAEA lines. Plant heights of five TG lines at the VT stage were lower by 55–82% compared with control plants (Fig 3A). Maximum extension rates were also lower in all TG lines (~ 9.1–17.6 cm/week) than in the controls (~ 25.8 cm/week) (Fig 3C) and the maximum rate occurred when the TG plants were significantly shorter (at 25–44 cm plant height) than the controls (at 76.9 cm plant height) (Fig 3D).

In contrast, the four TQ plants showed no significant difference in total plant height compared with controls (Fig 3B). Maximum extension rates were also similar between TQ and control plants (~23.5–26.3 cm/ week) (Fig 3E) and occurred when the TQ plants were approximately the same height (at 74.6–79.8 cm) as the controls (75.4 cm) (Fig 3F).

### Leaf, internode and epidermal cell morphology at the VT developmental stage

#### Internodes and leaves

**Fig 4:**
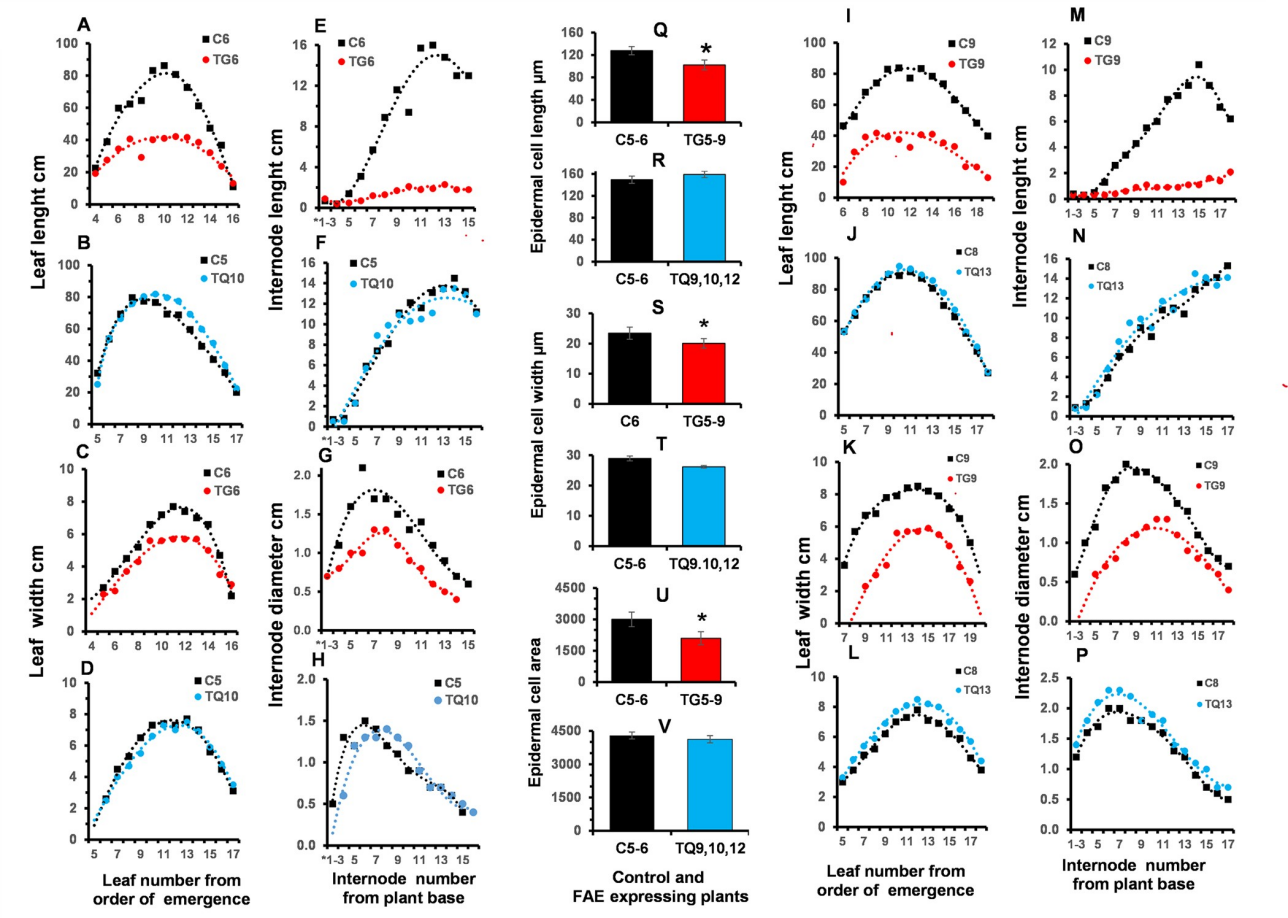
Leaf, internode and epidermal cell morphology of two FAEA expressing TG and TQ plants at the VT developmental stage. Leaf length **(A,B,I,J)**, maximum leaf width **(C,D,K,L)**, internode length **(E,F,M,N)** and internode diameter **(G,H,O,P)**, average epidermal cell length (**Q,R)**, width **(S,T)** and area **(U,V)** of the abaxial epidermis of fully expanded leaves of control and apoplast FAEA expressing plants TG6 **(A,E,C,G)**, TG9 **(I,M,K,O)**, TQ10 **(B,F,D,H)** and TQ13 **(J,N,L,P)**, and corresponding control plants (C6,C5,C8,C9) at the VT developmental stage. Mean ± seem (n = 10 replicates of 10 sections per sample, 1cm from the leaf tip, in rows adjacent to the leaf midrib) of plants TG 5–9, TQ9,10 and 12 and controls C5-6. * indicate significant differences (Tukey’s a = 0.001) among means.

TG plants were found to have shorter internodes and leaves compared with the controls throughout the V6-VT developmental stages, resulting in either extreme or semi-dwarfed plants. At the VT stage, plants TG6 and TG9 had 50 to 90% shorter and up to 62% thinner (Fig 4E and 4M) internodes than the controls (Fig 4G and 4O). Leaf lengths were also reduced by up to 78% (Fig 4A and 4I) and leaf widths by up to 65% (Fig 4C and 4K) compared with controls (Fig 4).

By contrast, no significant differences were found in the same four parameters in plants TQ10 and TQ13 (Fig 4B, 4F, 4D and 4H) compared with controls (4J, 4N, 4L and 4P). These phenotypes found for plants TQ10, TQ13 at the VT stage were also observed at the V6, V9 and V15 stages of development.

#### Epidermal dimensions and cell number

To establish whether the shorter leaves in the TG lines were a result of reduced cell expansion, we examined leaf anatomical features. The mean epidermal cell length of TG plants was 20% shorter than the control cells (P < 0.0001) (Fig 4Q) and varied from 69 to 115 μm in leaves of TG plants and 127–170 μm in control leaves. The epidermal cells of TG plants were also 14% narrower than control cells (Fig 4S) (P < 0.0001).

The significantly reduced mean cell length and width resulting in a 30% smaller epidermal cell area (Fig 4U) (P < 0.0001), could suggest a reason for the shorter, narrower leaves observed in the TG plants. However, preliminary epidermal cell number data in leaves of two TG plants revealed a significant 14% (P < 0.0023) and 20% (P < 0.0001) increase in cell numbers per unit area at the leaf tip, compared to control leaves (S7A Fig).

By contrast, measurements of epidermal cell size in TQ plants showed no significant differences (P = 0.1795; P = 0.2745 and P = 0.3718 for length, width and area respectively) from the controls (Fig 4R, 4T and 4V).

### FAEA activities in individual leaves and internodes of TG and TQ plants at four stages of development (V6 to VT)

#### FAEA activity in leaves

**Fig 5:**
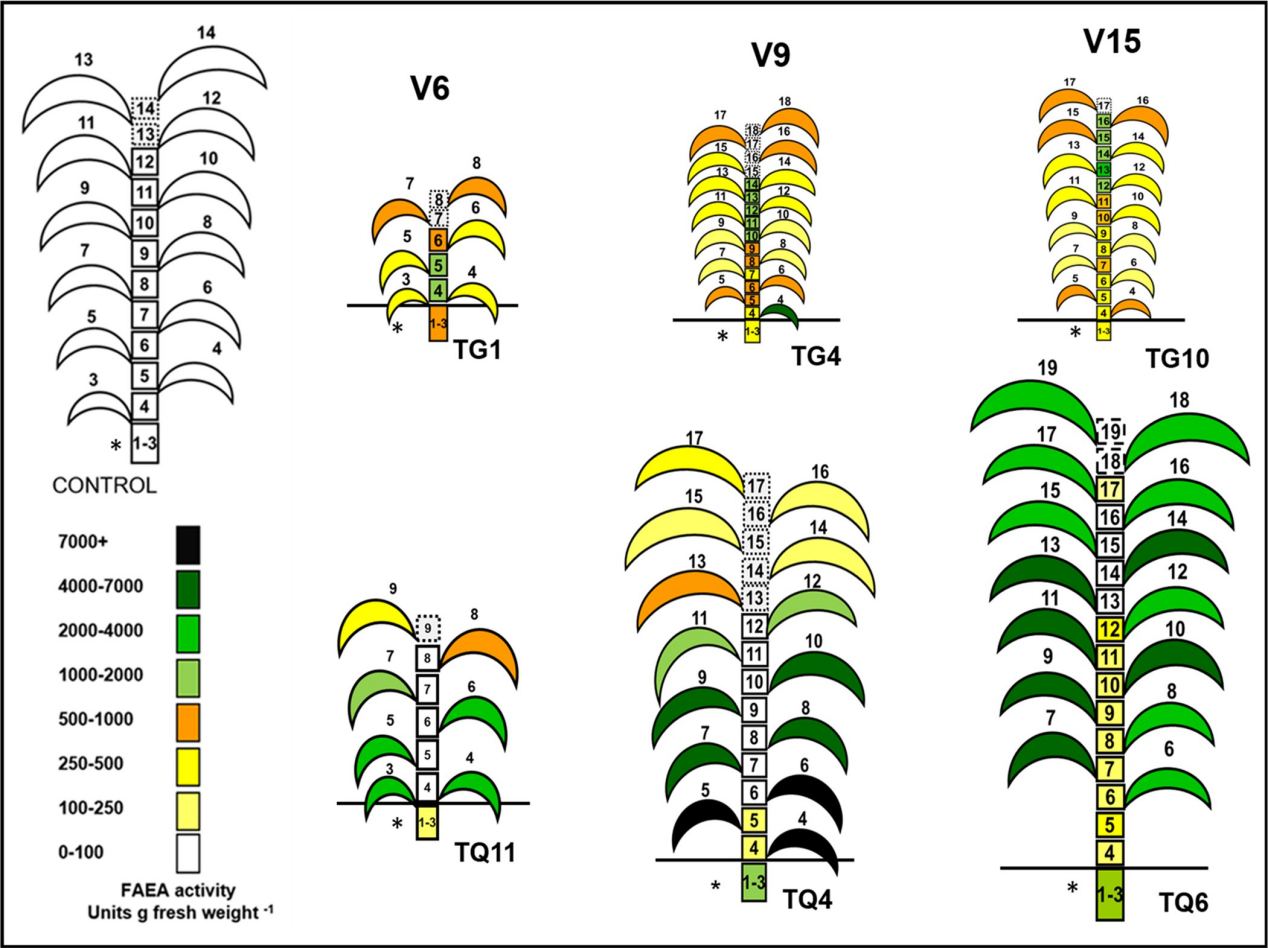
Comparison of overall FAEA activity in individual leaves and internodes of TG and TQ plants at three stages of development (V6 to V15). FAEA activity in leaves and internodes at V6, V9 and V15 developmental stages for TG1 and TQ11, TG4 and TQ4 and TG10 and TQ6. * indicates the fused internodes 1–3 or 1–4 and dotted lines refer to internodes that are not yet visible.

**Fig 6:**
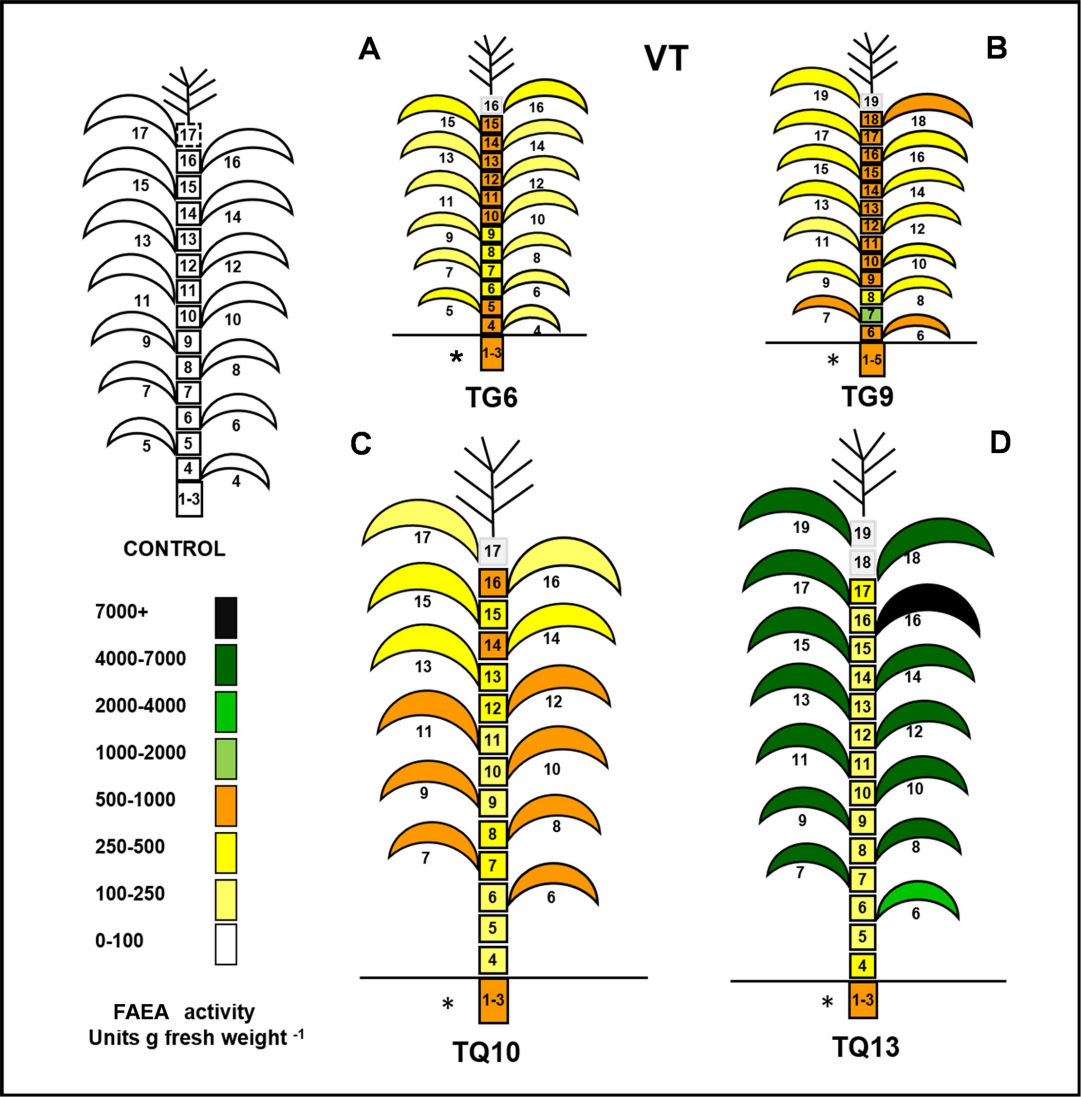
Comparison of overall FAEA activity in individual leaves and internodes of TG and TQ plants at VT stage of development. FAEA activity in leaves and internodes at VT developmental stage for TG6, TG9 and TQ10 and TQ13. * indicates the fused internodes 1–3 and dotted lines refer to no data internodes.

FAEA activities were found to vary with leaf age and between TG and TQ transgenic lines. In TG lines, FAEA expression started at an early stage of plant development, in very young leaves. During development, FAEA activity tended to be higher in younger and not fully expanded leaves (leaves 7–9 at V6, 10–17 at V9 and 15–17 at V15) (Fig 5 and S8A(a-c) Fig) and in younger leaves (16–17), at the VT stage, compared with fully developed and older leaves (Fig 6A and 6B). A comparison of the total FAEA per internode or leaf between TG and TQ plants at V6-V15 developmental stage shows that nearly all the FAEA activity (93–98%) was concentrated in the leaves of TQ plants, whereas in TG plants at the VT stage 61–72% of the FAEA activity was concentrated in the internodes (S8 Fig). Overall, FAEA activities were generally much higher in TQ lines than in the TG lines. However, in contrast to TG plants where FAEA activities were higher in younger leaves, FAEA activities in TQ plants were higher in older leaves and significantly lower in younger, not fully expanded leaves (for example leaves 7–9 at V6, leaves 11–17 at V9, as shown in Fig 5 and S8A(a,b) Fig, and leaves 14–17 at VT (S8B(g,h) Fig).

### FAEA activity in internodes

In maize internodes, cell elongation is initiated only after reproductive development begins [22]. This occurs initially and mainly by the expansion of internodes 7, 8 and 9, initially in coordination; with internodes 5 and 6 only expanding slightly. As a result, internodes 1–4 remain compact and below ground level, while the upper internodes expand at a slower rate but for longer period. It was found that in the TG plants, FAEA was expressed at all the developmental stages tested, starting well before the period of rapid internode elongation was initiated.

FAEA expression occurred in all internodes including the stage before rapid elongation (i.e. internodes 6 (at the V6 stage), 9–12 (at the V9 stage) and 16–17 (at the V15 stage) respectively. Fig 5 (which shows the overall distribution of FAEA activity in the plants) and S8A Fig (d-f which shows the corresponding data).

In contrast to this, except for the high levels of activity in the fused internodes 1–4, which do not undergo expansion, no significant FAEA expression was detected in the internodes of TQ plants at early stages (S8A(d-f) Fig). It was only after the internodes of TQ plants had started the period of rapid elongation that FAEA activity reached levels higher than the controls, such as at the VT stage shown for plants TQ10 and TQ13 (Fig 6C and 6D and S8B(g,i) Fig).

An interesting observation was that in TQ plants at the VT stage of development 65 ± 2% of the biomass and 21 ± 15% of FAEA activity was in the internodes, the remainder being in the leaves, whereas in TG plants at VT 46 ± 8% of the biomass and 60 ± 2% of FAEA activity was is in the internodes, with the remainder being in the leaves. Furthermore, in both TQ and TG plants at the VT stage of development over 90% of the stem biomass was found in internodes 6 to 16.

### Distribution of FAEA activity in TG and TQ internodes

**Fig 7:**
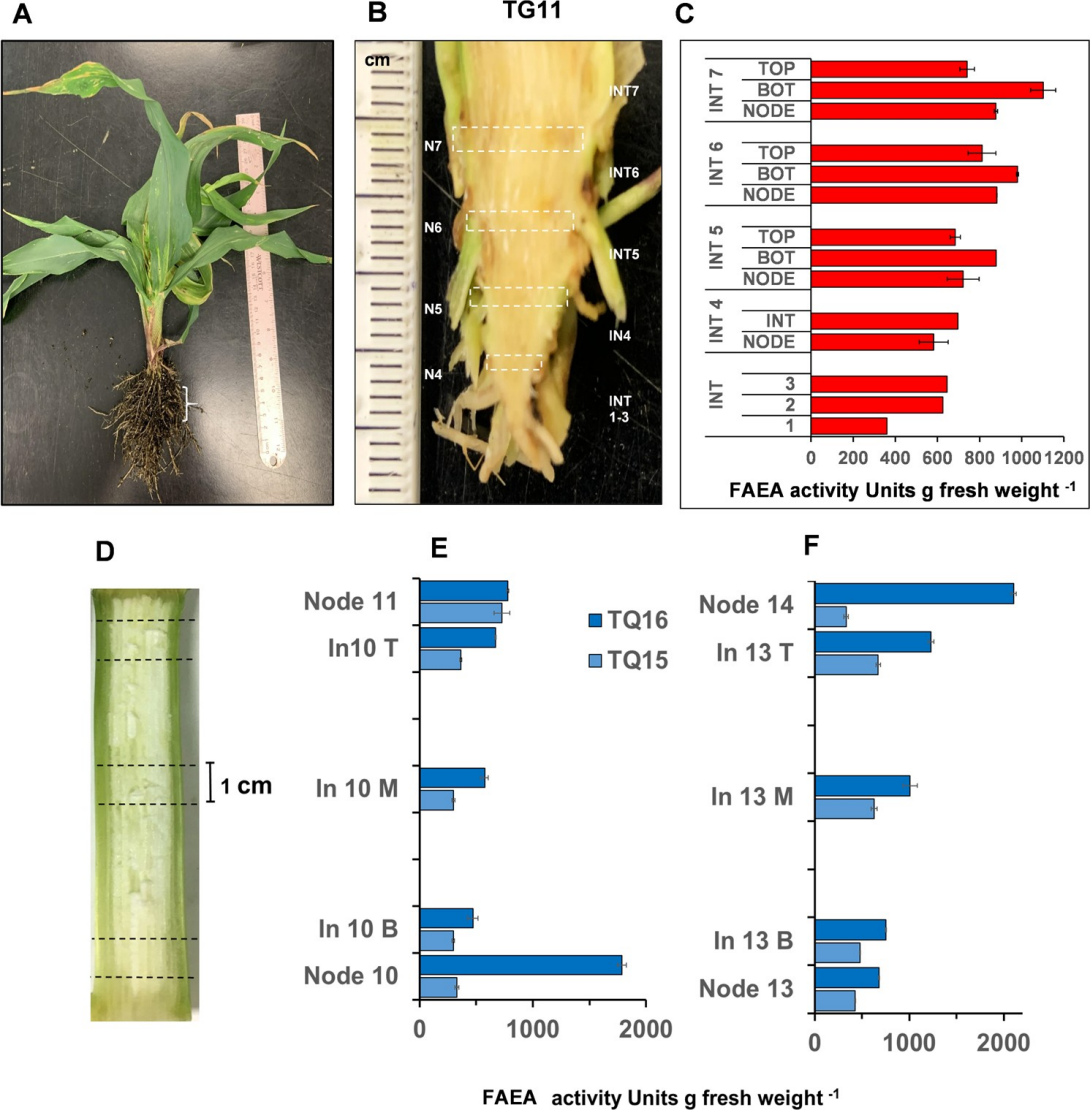
Distribution of FAEA activity in TG and TQ internodes. Dwarf mutant phenotype of plant TG11 at the V13 developmental stage **(A)**, and the internode phenotype **(B)**. FAEA activity levels in sections of fused internodes 1–3 and in nodes and internodes 4–7 **(C)** and FAEA activity in different regions of fully expanded internodes 10 of plant TQ15 and TQ16 (**E,F**) at the VR developmental stage and the internode phenotype **(D)**.

In maize, as in other Gramineae each internode provides a developmental gradient between nodes with four distinct regions, which have been described previously (Scobbie et al. 1993; Kend et al. 1998) [21, 23]. To establish if FAEA activities vary in nodes and along these regions, internodes were separated in sections and FAEA activities quantified. Because the length of internodes in TG plants were extremely short (Fig 7A and 7B), they were separated into base and top regions and the nodes (Fig 7B). FAEA expression was detected in all nodes and internode regions, with higher activity in the meristematic region (base) compared to the maturation zone (top) of the internode. FAEA activities in nodes and maturation zone were of similar magnitude and higher in younger internodes compared to older ones (Fig 7C). As internode in TQ plants were of normal length, 1 cm sections were taken from the base, middle and top parts of the internode, in addition to nodes for determining FAEA activities. While the level of FAEA expression varied between nodes, the level of FAEA activities were higher at the top of internodes compared to the middle and basal parts.

### Chemical phenotypes of FAEA expressing plants

#### Levels of ester-linked HCAs in internodes of TG and TQ plants at the VT stage of development

**Fig 8:**
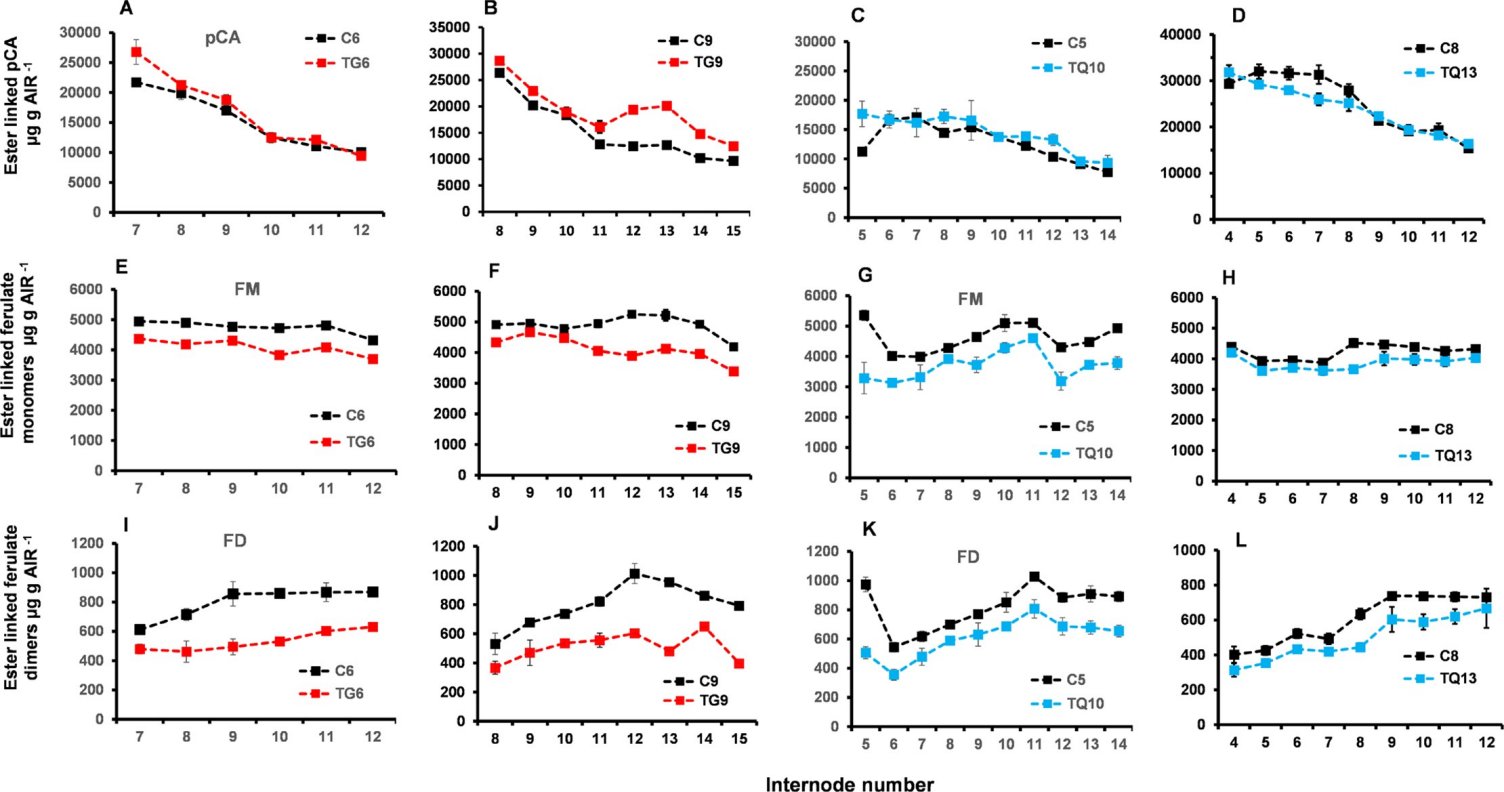
Levels of ester linked HCAs of selected internodes at the VT stage of development of two FAEA expressing TG and TQ plants. Ester linked *p*-coumaric acid (*p*CA) **(A-D)**, ester linked ferulate monomers (FM) **(E-H)** and ester linked ferulate dimers (FD) **(I-L)** of plants TG6 and control C6 **(A,E,I)**, TG9 and control C9 **(B,F,J),** TQ10 and control C5 **(C,G,K)** and TQ13 and control C8 **(D,H,L)**. Monomers; trans ferulic acid and cis-ferulic acid Dimers: 5–5' diferulic acid, 8-O-4' diferulic acid, 8–5 diferulic acid benzofuran form. Mean ± sem (n = 3).

The levels of ester-linked cell wall *p*-coumaric acid in internodes of TG and TQ plants were similar to the controls and apparently unaffected by FAEA expression (Fig 8A–8D). The levels of ester-linked ferulate monomers and dimers measured in internodes of plants TG6, TG9 and of TQ10 and TQ13, were lower in all 4 lines compared with their respective controls (Fig 8E–8L), consistent with FAEA being expressed in these internodes at the VT stage in both TG and TQ lines.

There was a 9–19% reduction in ester-linked ferulate monomers in internodes 7–12 of plant TG6, a 6–25% reduction in TG9, and a 9–38% reduction in TQ10. For ester-linked ferulate dimers, reductions ranged from 22–42% in TG6 and 6–11% in TG9, and 16–48% in TQ10 and 8–29% in TQ13, when compared with the corresponding controls. The ratio between ferulate monomers and dimers was also higher for both TG and TQ lines compared with controls.

### Levels of total (ester+ether) linked HCAs of selected internodes and leaves of TG and TQ plants at the VT stage of development

**Fig 9:**
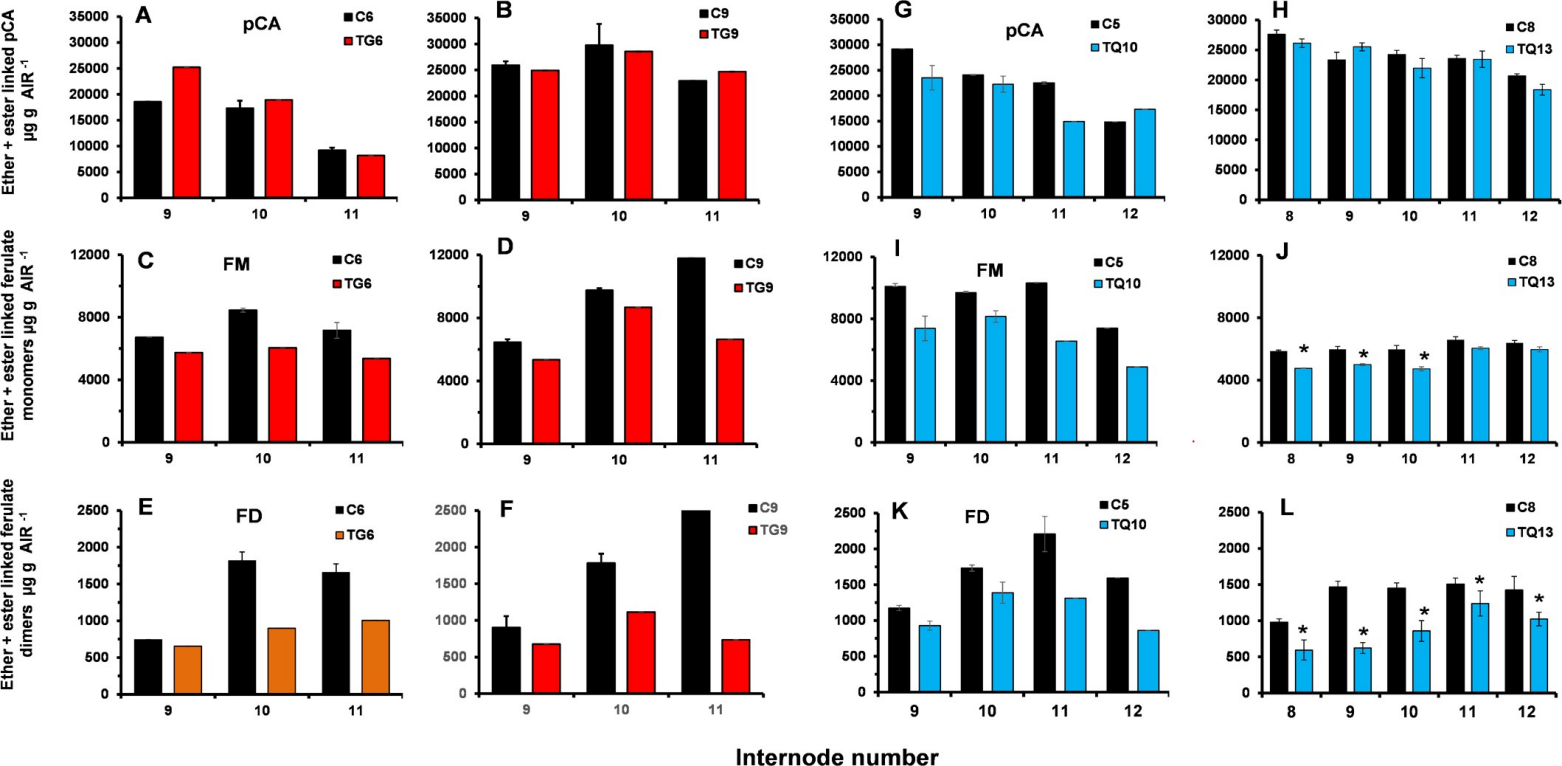
Levels of total ester+ether linked HCAs of selected internodes at the VT stage of development of two FAEA expressing TG and TQ plants. Ester linked *p*-coumaric acid (*p*CA) **(A,B,G,H)**, ester linked ferulate monomers (FM) **(C,D,I,J)** and ester linked ferulate dimers (FD) **(E,F,K,L)** of plants TG6 and control C6 **(A,C,E)**, TG9 and control C9 **(B,D,G),** TQ10 and control C5 and TQ13 and control C8 **(H,J,L)**. Monomers; trans ferulic acid and cis-ferulic acid Dimers: 5–5' diferulic acid, 8-O-4' diferulic acid, 8–5 diferulic acid benzofuran form. Mean ± sem (n = 3).

In TQ10 and TQ13 plants, total ester+ether linked *p*-coumaric acid levels were maximum in internodes 8–12 and in general higher in internodes of control plants (Fig 9G and 9H), while in internodes of TG6 plants ester+ether linked *p*-coumaric acid levels were not significantly different from controls (Fig 9A and 9B).

In internodes, the deposition of ester+ether-linked monomeric and dimeric ferulates were at a maximum in internodes 9–12 and were lower in both TG (Fig 9C–9F), and TQ lines (Fig 9I–9L) than in control internodes. However, the small number of replicates did not allow statistical analysis. In leaves of TG plants, although not statistically significant, except for leaf 16 (P > 0.0195*), the levels of ester+ether linked *p*CA were consistently lower in TG6 leaves 10–16 than in corresponding control leaves, while in plant TQ10 levels of ester+ether linked *p*CA were unaffected by FAEA expression (S10A and S10B Fig), despite higher levels of activity (Fig 6A).

Overall, in all leaves, the deposition of ester+ether linked monomeric (S10C and S10D Fig) and dimeric ferulates (S10E and S10F Fig) was statistically significantly lower in both TG6 and TQ10 lines than in controls.

### The relationships between FAEA activity and cell wall ferulate levels at earlier stages of development

**Fig 10:**
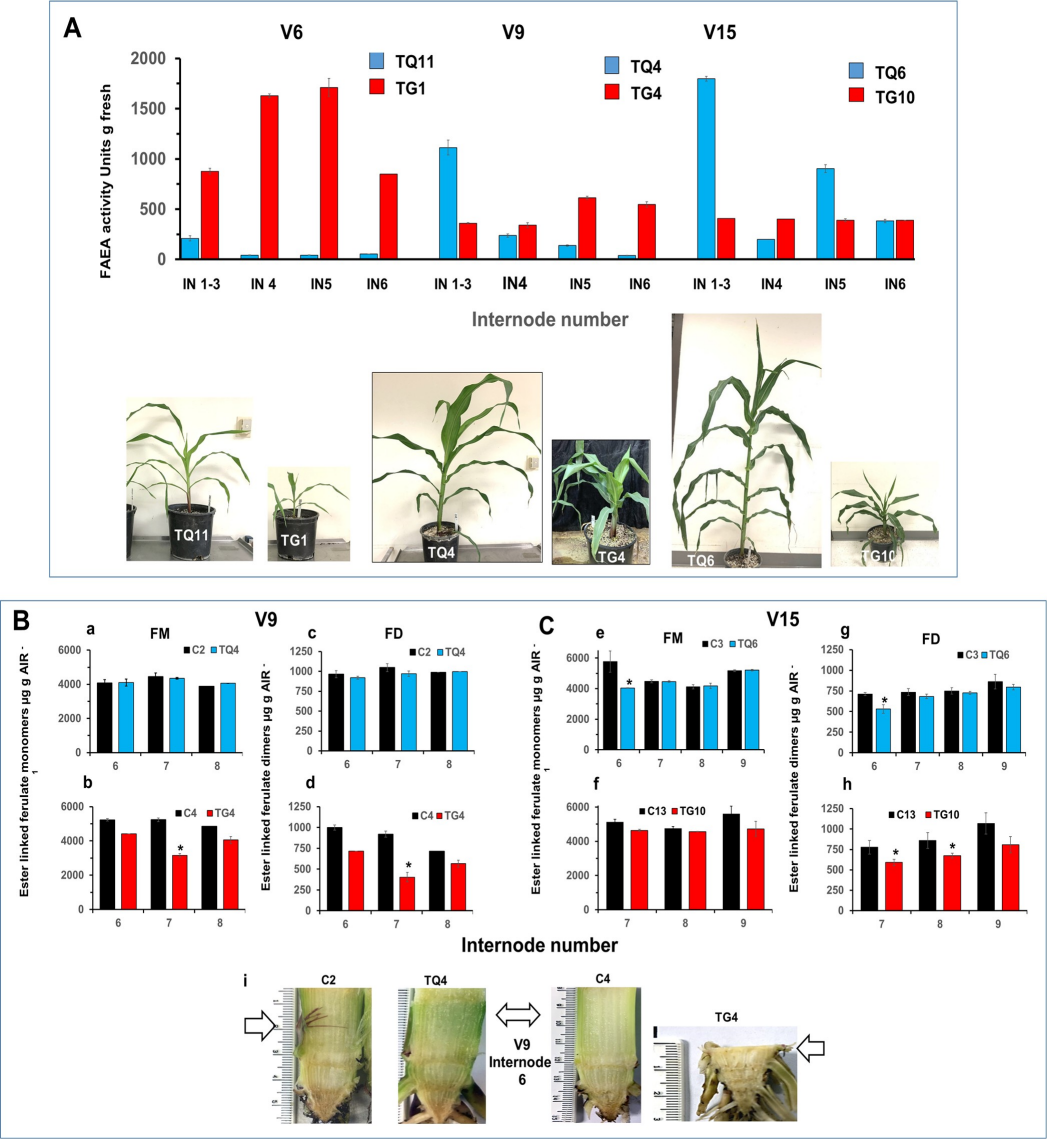
Relationships between FAEA activities and levels of ester-linked HCAs in internodes of TG and TQ plants at early stages of development. FAEA activity levels in internodes at V6, V9 and V15 developmental stages for selected TG and TQ plants and plants phenotypes **(A)**. Levels of cell wall esterified ferulate monomers **(a,b,e,f**) and dimers **(c,d,g,h)** in internodes of selected TG, TQ and control plants at V9 **(B) (a-d)** and V15 **(C) (e-h)** stages of development, and internode phenotypes for TG, TQ and corresponding controls at V9 (**i**). Monomers; trans ferulic acid and cis-ferulic acid. Dimers: 5–5' diferulic acid, 8-O-4' diferulic acid, 8–5 diferulic acid benzofuran form. * indicate significant differences from controls (Student’s α = 0.05).

FAEA activity in internodes of TG plants developed early during plant growth (by the V6 stage) and high levels were detectable in all internodes compared with FAEA activity in TQ internodes both on a gram fresh weight (Fig 10A) and on a whole internode basis (S9A Fig).

In order to determine if the lack of internode elongation in TG plants, but not in TQ plants was a consequence of the disruption of cell wall feruloylation by FAEA expression, before and/or during the period of normal rapid internode elongation, the levels of ester-linked ferulate monomers and dimers in the internodes of TG and TQ plants at the V9 and V15 stages were compared with controls (Fig 10B).

At the V9 stage, the levels of esterified ferulate monomers were lower by 15% to almost 40%, and ferulate dimers by 20–56% in internodes 6–8 of plant TG4 compared with its partner control plant C4 (Fig 10B). By contrast, there was no reduction in the level of ferulate monomers or dimers in the corresponding internodes of plant TQ4 when compared with the control C2 (Fig 10B). At this stage, FAEA activities were found in all internodes to include 6–8 of TG4 but not of TQ4 plants as previously shown (Fig 5 and S8A Fig) and internodes 6–8 had already started the period of rapid elongation.

At the V15 stage, high FAEA activities were observed in all internodes of plant TG10, but only in internodes 1–6 of plant TQ6 (Fig 10A and S8A Fig). In agreement with these results, the levels of esterified ferulate monomers were lower by 4–16% and ferulate dimers by 20–24% in internodes 7–9 of TG10 plants compared with plant C10 (Fig 10C).

In contrast, it was found that levels of ferulate monomers and dimers were lower (by 29% and 25%, respectively) only in internode 6 of plant TQ6 (Fig 10C). As expected, there was no difference in the level of ferulate monomers and dimers in internodes 7–9 of plant TQ6 compared with plant C3 (Fig 10C). As internode 6 had already passed through the period of rapid elongation by the V15 stage, FAEA expression and the changes in ester-linked ferulate levels observed in TQ6 plants had no effect on internode elongation, resulting in TQ plants being of similar heights to the control C3 plant.

### Levels of cell wall sugars (arabinoxylan and glucose) in selected internodes of TG and TQ plants at the VT stage

Changes in the composition of the cell wall in relation to their arabinoxylan and glucose content was determined in selected internodes of TG and TQ plants at VT stage of development.

**Fig 11:**
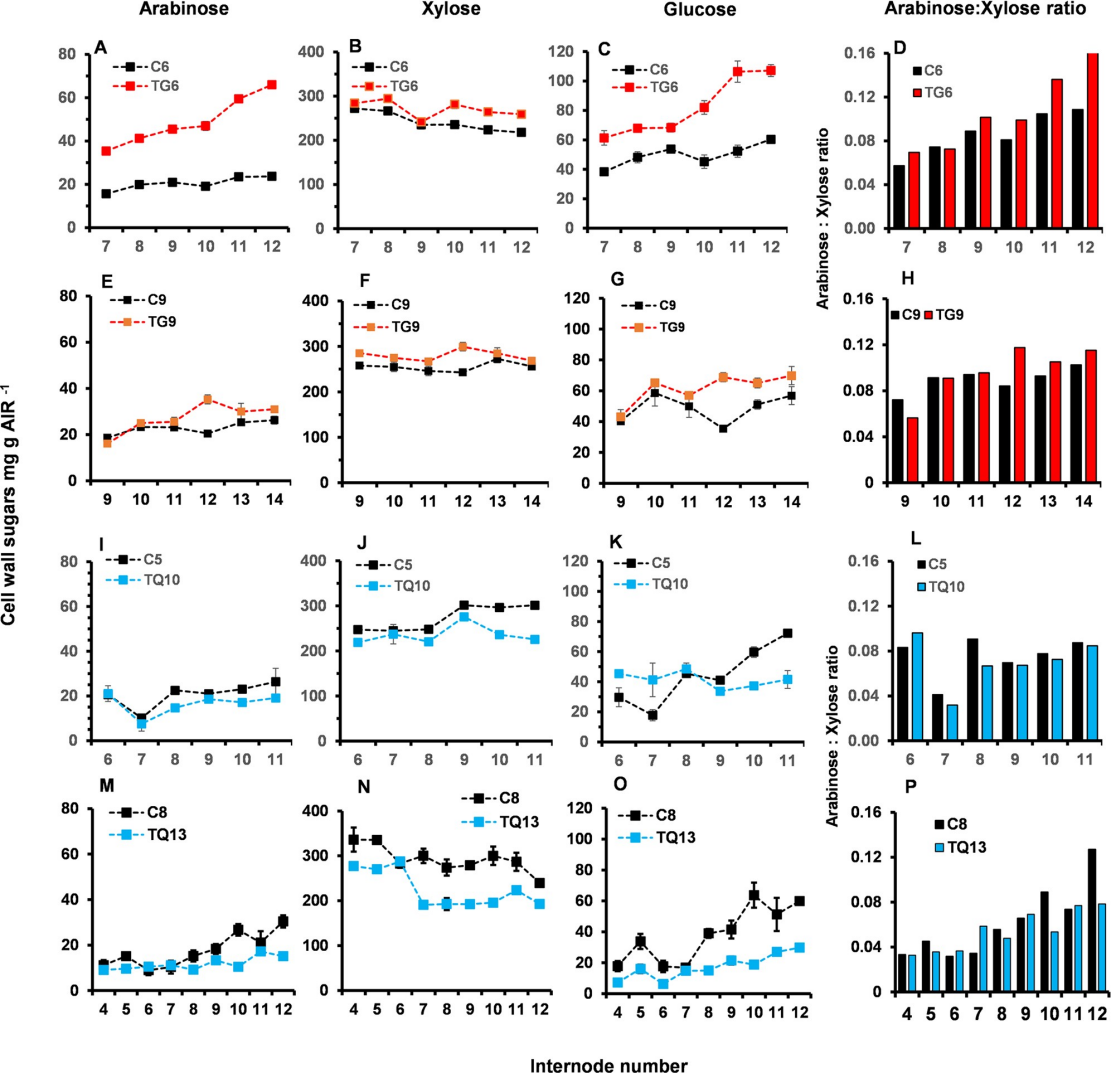
Levels of cell wall sugars (arabinoxylan and glucose) of selected internodes at the VT stage of development of two FAEA expressing TG and TQ plants. Levels of arabinose **(A,E,I,M),** xylose **(B,F,J,N),** and glucose **(C,G,K,O),** and the arabinose to xylose ratio, **(D,H,LP)** of TG6 and control C6 plants **(A-D)**, TG9 and control C5 plants **(E-H),** TQ10 and control C5 plants **(I-L)** and TQ13 and C8 control plants **(N-P)**. Mean ± sem (n = 3).

Internodes of the TG6 and TG9 lines contained between 7.7% and 78% more arabinose, between 3% and 19% more xylose and between 27% and 103% more glucose than control internodes (Fig 11). Pairwise comparisons between TG6 and control internodes showed significantly increased levels of arabinose, xylose and glucose, especially for internodes 10 (arabinose P < 0.0521; xylose P <0.0371 and glucose P < 0.0124), 11 (arabinose P < 0.0009; xylose P <0.0053 and glucose P < 0.0029) and 12 (arabinose P < 0.0009; xylose P <0.0011 and glucose P < 0.0006) (Fig 11A–11C). The higher percentage increase in levels of arabinose in TG lines compared to other sugars, is reflected in the increase in the arabinose: xylose ratio observed for most of TG internodes (Fig 11D and 11H).

A different trend was observed for the arabinose, xylose and glucose content of internodes of TQ10 and TQ13 plants which contained between 11% and 34% less arabinose, between 8% and 25% less xylose and between 17% and 42% less glucose than control internodes. Pairwise comparisons between TQ10 and control internodes showed significantly decreased levels of arabinose, xylose and glucose in internodes 10 (arabinose P < 0.0022; xylose P <0.0004; glucose P < 0.0057) and 11 (arabinose P < 0.0028; xylose P <0.0025 and glucose P < 0.01) (Fig 11I–11O).

### The relationship between FAEA activity, and ester-linked HCAs in internodes of senescing TQ plants

Part of the aim of expressing FAEA driven by a senescence enhanced promoter was to establish to what extent the levels of cell wall ferulates could be reduced in senescent maize stova, without adversely affecting plant development or biomass yield, and which would be suitable for subsequent post-harvest enzymatic degradation or simultaneous saccharification and fermentation (SSF). The FAEA activity, and levels of cell wall ferulate were therefore also determined at the completion of plant growth at the VR5 (dent) stage, when plant senescence is almost complete and stova could be harvested.

**Fig 12:**
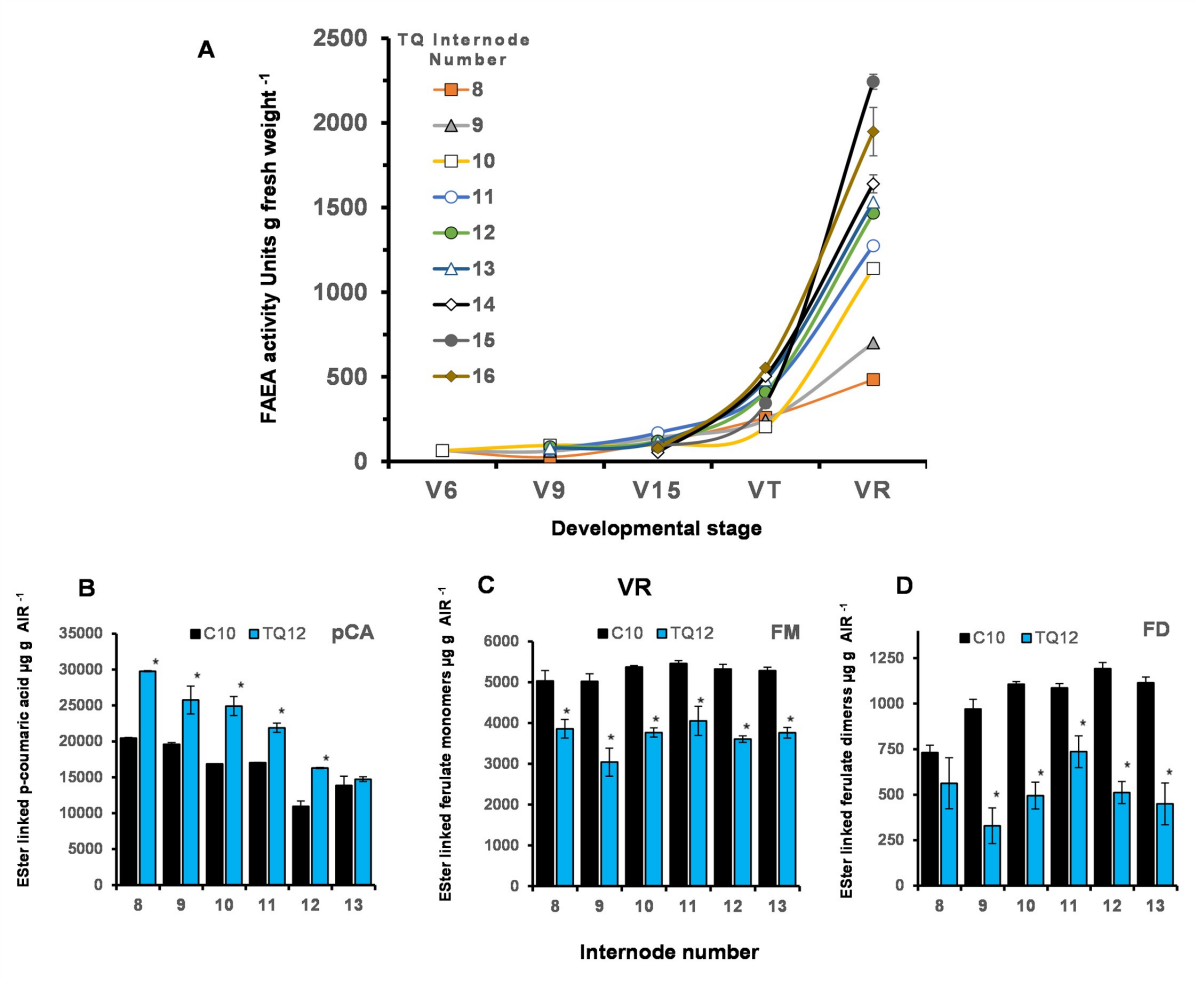
Cumulative FAEA activity from V6 to VR developmental stages and levels of ester-linked HCAs in internodes at the VR stage. Accumulative FAEA activities in internodes 8 to16 from V6 to VR developmental stages of plants TQ11 (V6), TQ4 (V9), TQ6 (V15), TQ10 (VT) and TQ12 (VR) **(A)**. Ester linked *p*-coumaric acid (*p*CA) **(B)**, ester linked ferulate monomers (FM) **(C)** and ester linked ferulate dimers (FD) (D), of plant TQ12 and control C10 at VR. Monomers; trans ferulic acid and cis-ferulic acid Dimers: 5–5' diferulic acid, 8-O-4' diferulic acid, 8–5 diferulic acid benzofuran form. Mean ± sem (n = 3) * indicate significant differences from controls (Student’s α = 0.05).

The FAEA activity in TQ internodes was found to be very low up to the VT stage of development but increased significantly at the VR stage in internodes 8 to 16 (which accounts for more than 75% of the stova biomass) (Fig 12A), and confirms that FAEA activity in internodes of TQ plants increases as plants begin to senesce. These significantly higher FAEA activities in TQ internodes at the VR stage resulted in a 23–39% reduction in ester-linked ferulate monomers across internodes 8 to 13 (Fig 12C) and a 23–66% decrease in ester-linked ferulates dimers (Fig 12D), compared with TQ plants at the VT stage (Fig 8G, 8H, 8K and 8L, respectively), but a 6–48% increase in the levels of *p*-coumaric acid compared with controls (Fig 12B).

## Discussion

Cell walls are complex structures that surround plant cells and serve several important functions in the life of plants, such as controlling the growth behavior of individual cells. While plant cell expansion is driven by turgor pressure, the control of this process relies on the mechanical properties of their walls. Modulation of the cell wall mechanical properties is achieved by a dual process of loosening and tightening of the primary cell wall which occurs by regulating the cell wall chemical composition (synthesis of new polymers) as well as its integration into the existing wall and the creation of new cross-links between wall polymers [2, 9].

Ferulate oxidative coupling of arabinoxylan sidechains constitutes a significant type of cross-link in grass cell walls which, for the points raised above, are expected to have a significant role in plant growth. The effectiveness of reducing the level of cell wall feruloylation in grasses by expression of a ferulic acid esterase from *Aspergillus niger* to different cell compartments has been established [34] and shown to work as a useful tool to study the direct effects of feruloylation on different plant processes [34, 37, 39].

In this study, the role of cell wall feruloylation in leaf development and during internode expansion was investigated in maize making use of plants expressing apoplast targeted *Aspergillus niger* FAEA placed under the control of either a constitutive or an inducible promoter. In all lines where FAEA was targeted to the apoplast under the constitutive promoter, FAEA expression started early and well before the internode fast elongation stage was initiated. When the cell wall HCA compositions in internode sections and leaves of these maize plants at V9, V15 and VT stages were quantified, it was confirmed that monomeric and dimeric ester-linked ferulate levels was reduced by over 26% and 42%, respectively. The levels of monomeric and dimeric ester+ ether-linked ferulates was also reduced at the VT stage by over 43% and 71% in internodes and by 55% and 51% respectively in leaves compared with controls. These TG plants had shorter and narrower leaves and internodes, and an overall dwarfed phenotype compared to controls. In addition, a small number of plants (approximately 20%) showed an extreme dwarf phenotype ranging from 8 to 12 cm maximum height at the VT stage which showed similar characteristics to the larger semi-dwarf plants in terms of disruption of vascular sclerenchyma, FAEA expression in leaves and internodes, reduced levels of some ester linked ferulates and increased levels of cell wall sugars and increased levels of cellulase mediated glucose release from the internode cell walls.

Together, these results show that the disruption of cell wall feruloylation by FAEA in the TG lines probably occurs at a very early stage of development, i.e. before the start of rapid internode expansion is initiated and affects the normal course of internode expansion and results in short internodes and dwarfed plants. However, it is clear that some internode expansion was possible in the semi-dwarf plants.

When under the inducible *Lm See1* senescence promoter, FAEA activity was not induced until after internodes had passed their period of rapid elongation and therefore there was no change in cell wall ferulates at this point and plants showed similar phenotypes to controls. FAEA activity in TQ lines occurring only after the rapid internode expansion stage was passed did not appear to prevent internode extension.

These findings strongly suggest that normal cell wall feruloylation is required for the normal process of internode expansion in maize. The effect of altering the level of cell wall feruloylation on plant growth and leaf morphology was recently studied in tall fescue by constitutively targeting FAEA expression to different cell compartments [39]. Reducing the level of cell wall feruloylation in leaves resulted in narrower and shorter leaves and overall lower growth rates compared to controls. In rice, suppression of UDP-arabinopyranose by UDP- arabinopyranose mutase resulted, in addition to reduced levels of arabinose, less ferulic and *p*-coumaric acid in their cell walls, dwarfism and infertility [49].

It could be argued that FAEA expression affects plant growth by releasing ferulates from the wall which could accumulate in the cells and potentially inhibit enzymes such as amylase and maltase, which are involved in seed starch metabolism. Inhibition of such enzymes would result in a reduced supply of glucose, and consequently, would negatively affect different physiological processes such as growth and development of germinating maize seedlings. It has been previously shown that ferulic acid-treated maize seedlings showed significant reduction in growth, where the activities of hydrolytic enzymes were negatively affected [50]. Although the level of starch degrading enzymes during seedling development was not measured here, TG seedlings up to V4 stage did not show much phenotypic difference compared with control so the accumulation of FAEA in cells of TG plants is unlikely.

Constitutive FAEA expression also resulted in significantly smaller leaf epidermal cells compared with control leaves and contrasted with plants expressing FAEA under the senescence promoter which had epidermal cells of normal sizes, indicating that FAEA expression early in leaf development can arrest normal cell growth. These results are consistent with data reported in *Festuca* [39] which showed that constitutive expression of FAEA to apoplast and Golgi also resulted in smaller leaf epidermal cells. The authors suggested that these findings were likely the result of early cessation of ferulate deposition and dimerization which also occurred at a lower level in FAEA expressing plants compared to controls. It has also been shown that slender barley mutants had longer leaf length and longer epidermal cells compared to control plants [51]. Although the mechanisms for barley mutants and FAEA expressing plants are different, these results do suggest that changes in individual cell length, can result in changes in leaf length. In addition to smaller epidermal cells, significant increased cell density implies that epidermal cells in TG lines had increased cell wall density. This could explain the lack of a statistically significant difference in ester-linked cell wall ferulates observed among some of the samples from TG plants. As internode expansion is a combination of cell division and elongation, we could speculate that the increase in cell number would work as a compensatory mechanism to compensate for reduced epidermal cell sizes.

In addition to shorter and narrower leaves, constitutive FAEA expression in maize also resulted in thinner walled enlarged cell walls of the sclerenchyma, the supportive tissue between xylem and epidermis, and less rigid tissue, but with no evidence of xylem collapse.

There are some possible explanations for the thin walled enlarged cells of the sclerenchyma and less rigid internode sections of TG plants. One possibility is that the reduced levels of ferulates in the walls of the TG plants, as confirmed by reduced level of both ester and ether+ester linked diferulates, may lead to reduced cross-linking between arabinoxylan and lignin, leading to a less compact cell wall structure with reduced adherence to the primary cell wall. A second possibility is that the increase in cell wall arabinoxylan content in TG plants, which are hydrophilic, leads to higher water absorption and therefore swollen cells, which then results in a more water-permeable wall, which is prone to fall apart as observed when sectioning. A third possibility is that ferulates have a crucial role to play in cell-to-cell adhesion and alterations in the levels of ester linkages mediated by ferulic acid between middle lamella and primary cell wall in TG plants, from early stages in plant development, will affect this process resulting in less rigid internode sections. Previous studies involving sugar beet and Chinese water chestnut showed that removal of ferulate dehydrodimers by diluted alkali, resulted in vortex-induced cell separation [31, 32] supporting the notion that the degree of thermal stability of cell-to-cell adhesion could be related to the presence of ferulates cross-linking the xylan backbone of AX and the arabinose of RG-I.

It was also observed that constitutive FAEA expression resulted in increased levels of cell wall arabinoxylan and glucose. Changes in cell wall feruloylation as a result of FAEA expression that alters cell wall architecture, might be triggering compensatory mechanisms in TG plants, to compensate for the reduction in the levels of both ester and ether+ester linked diferulates. Such compensatory mechanisms in plants has been suggested by Hu et al. (1999) [52] based on the observation that the down regulation of the 4-coumarate: coenzyme A ligase (4CL) gene in *Populus* resulted in trees with reduced level of lignin which was compensated for by an increase in cellulose. Vermerris et al. (2010) [53] also reported that the reduced lignin content in *bm2-bm4* maize double mutants was compensated by an increase in hemicellulose levels. The *xax1* rice mutants which had reduced xylan and a lower degree of diferulate cross-links, also exhibited ~10% increase in terminal arabinose [54]. More recently Pellnny et al. (2020) [55] have shown that knocking down expression of three *Ta*IRX9B (orthologs of *Arabidopsis* irx9) genes in wheat endosperm resulted in reduced levels of AX and increased ferulate dimerization. These observations, which are in line with the data presented here, points to a compensatory mechanism in plants that indicates a level of interdependence between different groups of cell wall components.

There are several possible mechanisms by which the cell wall feruloylation process can affect cell wall expansion and plant growth. One relates to the dual process of loosening and tightening of primary cell wall components which controls growth in an immature plant cell [15]. The synthesis and integration of new polymers and the formation of cross-link between these polymers are an essential part of the growth process. As ferulate cross-linking AX are a significant type of cross-link in grasses [56], they are likely to be essential for growth of cells by providing cell walls with mechanical strength to sustain the pressure put upon the wall during growth. O’Neill et al. (2001) [57] have suggested that cross-linking of RGa-II by borate is essential for *Arabidopsis* cell growth by providing the cells with mechanical strength. This has been supported by *Arabidopsis* mur-1 mutant that exhibited a 30% reduction in RG-II dimerization and a dwarfed phenotype. Although cell wall cross-link mechanisms in grasses and in dicots involve different chemical compounds, these results do suggest that the cell wall cross-link process can provide cells with mechanical strength to sustain the pressure that is put upon cell walls during plant growth and as such is likely to be essential for normal cell wall expansion and plant growth. Another possible mechanism would involve FAEA disrupting the structure of the new cell wall following deposition of cellulose microfibrils. A generally cited hypothesis is that radial constriction of cell expansion by orientation of new cell wall around the cell will only allow the cells to elongate, under turgor pressure. Cellulose microfibril orientation is typically perpendicular to the main axis of growth [2] although axillary aligned cellulose microfibrils in elongating parenchyma cells of Popular has been reported [58].We hypothesize that ferulate cross-linking is involved in this process and FAEA expression would disrupt the structure of the new cell wall following deposition of cellulose microfibrils, at the base of the node. This would arrest cell elongation which would instead increase the cell diameter, as possibly seen in the toluidine stained internode sections of TG plants. If this hypothesis is correct, then feruolylated AX would need to be deposited and dimers need to be present early during primary cell wall deposition in the elongation zone of the internode. And indeed, both the primary cell wall and solubilized extracts (protoplasmic faction) of cultured maize cells have been shown to contain ferulate dimers [59], and at least some hemicelluloses, such as xyloglucans, can interact with the surface of cellulose and form cross-links between adjacent microfibrils [60]. It has also been proposed that the reorientation of cellulose microfibrils is driven by turgor pressure induced wall elongation and that cross-linking by secreted wall components such as hemicelluloses could be expected to occur predominantly between closely spaced cellulose microfibrils [61].

Earlier *in-planta* apoplast targeted expression of FAEA in leaves of *Festuca* [39] and in maize leaves and internodes (this work), when constitutively driven by the actin promoter, disrupted feruloylation of growing cells, affecting plant development and resulted in a significant reduction in plant biomass.

In contrast to this, *in-planta* expression of FAEA under the *Lolium See1* senescence enhanced promoter [62] produced plants with similar phenotypes and biomass compared with controls. It has been previously shown that where FAEA was under the control of this senescence enhanced promoter, transgenic *Festuca* plants exhibited similar phenotypes compared to controls [34]. The similar maize phenotype was a result of FAEA being expressed in internodes later in plant development, after they had passed the stage of fast internode expansion, and as a result cell wall feruloylation levels were altered only later after cell wall formation, with no major impact on plant growth and with a major positive effect on cell wall degradability. Also, given the high levels of FAEA in the leaves and low levels in the internode of TQ plants and the reverse in TG plants, it is not surprising that little cell disruption can be seen in TQ internode sections. Cellular apoptosis of parenchyma and xylem cells, mediated by serine proteases, occurs concurrent with secondary cell wall biosynthesis as the internodes expand [63, 64] and may be responsible for triggering low levels of FAEA expression in internodes of TQ plants where FAEA is under the control of the *See1* protease promoter. Although the vascular system of the leaf midrib of TQ plants was not analyzed, the fact that FAEA activity in the midrib was much lower compared to the leaf blade (S7B Fig) indicates that the vascular system of the midrib of TQ leaves may not be affected by FAEA expression in the leaf lamina. An interesting observation was the significant increase in *p-*coumaric levels in TQ internodes at the VR stage. This could also be explained as a compensation mechanism in response to reduced ester linked ferulates, specially ferulate dimers and probably ether ferulates. Silencing of the BAHD acetyl-CoA transferase ortholog (*Sv*BAHD01) in *Setaria viridis* resulted in significant reductions of ferulates in stem cell walls and a significant increase in *p*CA in leaves but only a small increase in stems [65]. While ferulates cross-link adjacent xylan chains to one another (ester linkages) [9], the presence of ether linkages between ferulates and lignin has been suggested to work as the nucleation site for lignification in grasses and to mediate lignin-polysaccharide cross-linking, adding strength to the cell wall [33, 66, 67]. In addition to *p*-coumaric acid coupling propensity, *p*-coumarates work as a radical transfer system, acylating lignin monomers, specially sinapyl alcohols, assisting with lignin polymerization [67]. Thus, the increase in *p-*coumaric in TQ lines could be working to compensate for the reduced level of ferulates, which are also crucial participants in lignification.

## Conclusions

There are two aspects to this work we wish to communicate, firstly the inhibitory effect of constitutive FAEA expression on internode expansion shown in the TG plants, which can result in a shortening of internodes in some lines and semi-dwarf plants and in some cases extreme statute mutants, indicating the importance of normal cell wall feruloylation in the early stages of cell and internode elongation.

And secondly the successful production of TQ maize lines expressing FAEA under a senescence enhanced promoter with little effect on plant development or biomass yield but with lower levels of cell wall ferulates throughout the stova of mature plants. The effects of this reduction in cell wall ferulates on stova digestion, saccharification and ethanol fermentation will be reported in a separate publication.

## Supporting information

S1 Fig Phenotypes of FAEA expressing TG and TQ plants at four stages of development. Plants TG1+C1 and TQ11+C10 at the V6 stage **(A),** TG4+C4 and TQ4+C2 at the V9 stage **(B)**, TG10+C10 and TQ6+C3 at the V15 stage **(C)** and TG6+C6 and TQ10+C5 at the VT stage of development **(D)**.
(TIF)

S2 Fig Examples of the range of TG stature mutants. Nine plants from 127cm to 8cm high at maturity, and internode sections stained with phloroglucinol or toluidine blue, showing the relationship between plant height and the extent of cellular disruption of the vascular tissues (A-C).
(TIF)

S3 Fig Properties of three extreme dwarf FAEA expressing TG mutants. Plant phenotypes **(A)**,transverse sections of internodes 2, 3, or 4 stained with either toluidine blue or phloroglucinol **(B)**, FAEA activity of leaves internodes 1–4 and tassel **(C),** levels of cell wall HCAs of internodes 1–4; *p*-coumaric acid (*p*CA), ferulate monomers (FM) and ferulate dimers (FD) **(D)**, cell wall sugars of internodes 1–4; arabinose (Arab), xylose (Xyl) and glucose (Glu) **(E),** and cellulase mediate release of total sugars from internodes 1–4 of the three mutant plants **(F)**.
(TIF)

S4 Fig Three T_0_ stature mutants of maize transformed with *p*INH1Δ expressing vacuolar targeted FAEA under the rice actin promoter. Plant phenotypes (**A**), FAEA activity in leaves compared with controls **(B)** and levels of FAEA activity of different tissues from a single plant TΔ4 (**C**). Tissues are ranked by FAEA activity. Mean ± seem (n = 3).
(TIF)

S5 Fig Cell morphology phenotypes of internode sections at the VT stage of development of FAEA expressing TG and TQ plants. Transverse sections through the central vascular bundles of internode 6 of apoplast FAEA expressing plants. TG6 **(A,E)** and TQ10 **(C,G)**, and control plants C6 **(B,F)** and C5 **(D,H)**, grown under greenhouse conditions to the VT tasselling stage of development, stained with toluidine blue **(A-D)** or phloroglucinol **(E-H)**.
(TIF)

S6 Fig Stem internode section and plant morphological phenotypes of FAEA expressing TG and TQ plants at the VT stage of development. Transverse sections through the central vascular bundles of internode 6 of and phenotype of apoplast FAEA expressing plants TQ13 and TG9, and control plants C8 and C9, grown under greenhouse conditions to the VT tasselling stage of development, stained with toluidine blue.
(TIF)

S7 Fig Epidermal cell numbers in TG plants and comparison of FAEA activities in TQ leaf lamina and leaf midribs. Number of epidermal cells mm ^-2^ in terminal leaves of VT stage plants TG9 and TG12 compared with corresponding control plants C15 and C16. The number of cells was counted within 600,000 μ^2^ areas randomly selected from 10 leaf zones from 2 leaves per line (**A**). FAEA activity in leaf lamina and midribs of leaves 4 to 8 of V6 plants TQ17 and TQ 18 compared with a control plant C17 (**B).** Mean ± sem (n = 3).
(TIF)

S8 Fig Comparison of FAEA activity in individual leaves and internodes at different stages of development (V6 to VT) of TG and TQ plants. FAEA activity levels in (**A**) leaves **(a-c)** and internodes **(d-f)** at V6 **(a,d),** V9 **(b,e)**, V15 **(c,f),** and **(B)** leaves **(g-h)** and internodes **(i-j)** at VT **(g-j)** developmental stages for TG1 and TQ11 **(a,d)**, TG4 and TQ4 **(b,e),** TG10 and TQ6 (**c,f**),TG6 and TQ10 (**g,i**), TG9 and TQ13(**h, j**), and corresponding control plants (C1-C14, C2-C4,C3-C13, C5-C6 and C8-C9). Values for controls are the means ± sem (n = 6) of the TQ and TG control plants and values for TG ad TQ plants are the means ± sem (n = 3). * indicates the fused internodes 1–3.
(TIF)

S9 Fig Total FAEA activity per leaf and per internode of TG and TQ plants at V6 to VT stages of development. FAEA activity in internodes **(A-C, G-I)** and leaves **(D-F, J-L)** at V6 **(A,D),** V9 **(B,E),** V15 **(C,F)** and VT **(G-L)** developmental stages for TG1 + TQ11 **(A,D)**, TG4 + TQ4 **(B,E)**, TG10 + TQ6 **(C,F)** and for TG6+TQ10 **(G,J)**, TG9,+ TQ13 **(H,K)**, and TG7 + TQ9 **(I-L)**. *indicates the fused internodes 1–3.
(TIF)

S10 Fig Total (ester + ether linked) HCAs of cell wall AIR of selected leaves of FAEA expressing TG and TQ plants at VT stage. Levels of *p*-coumaric acid **(A,B)** ferulate monomers **(C,D)** and ferulate dimers **(E,F)** of selected leaves of plants TG6 + C6 control **(A,C,E)** and TQ10 + C5 control **(B,D,F).** Single determinations or means ± sem (n = 3). * indicate significant differences from controls (Student’s α = 0.05).
(TIF)
