# Supplementary figures and images for: Probing the role of cell wall feruloylation during maize development by differential expression of an apoplast targeted fungal ferulic acid esterase
Source: PLoS One. 2020 Oct 9;15(10):e0240369. doi: 10.1371/journal.pone.0240369 (PMC7546508; doi:10.1371/journal.pone.0240369)

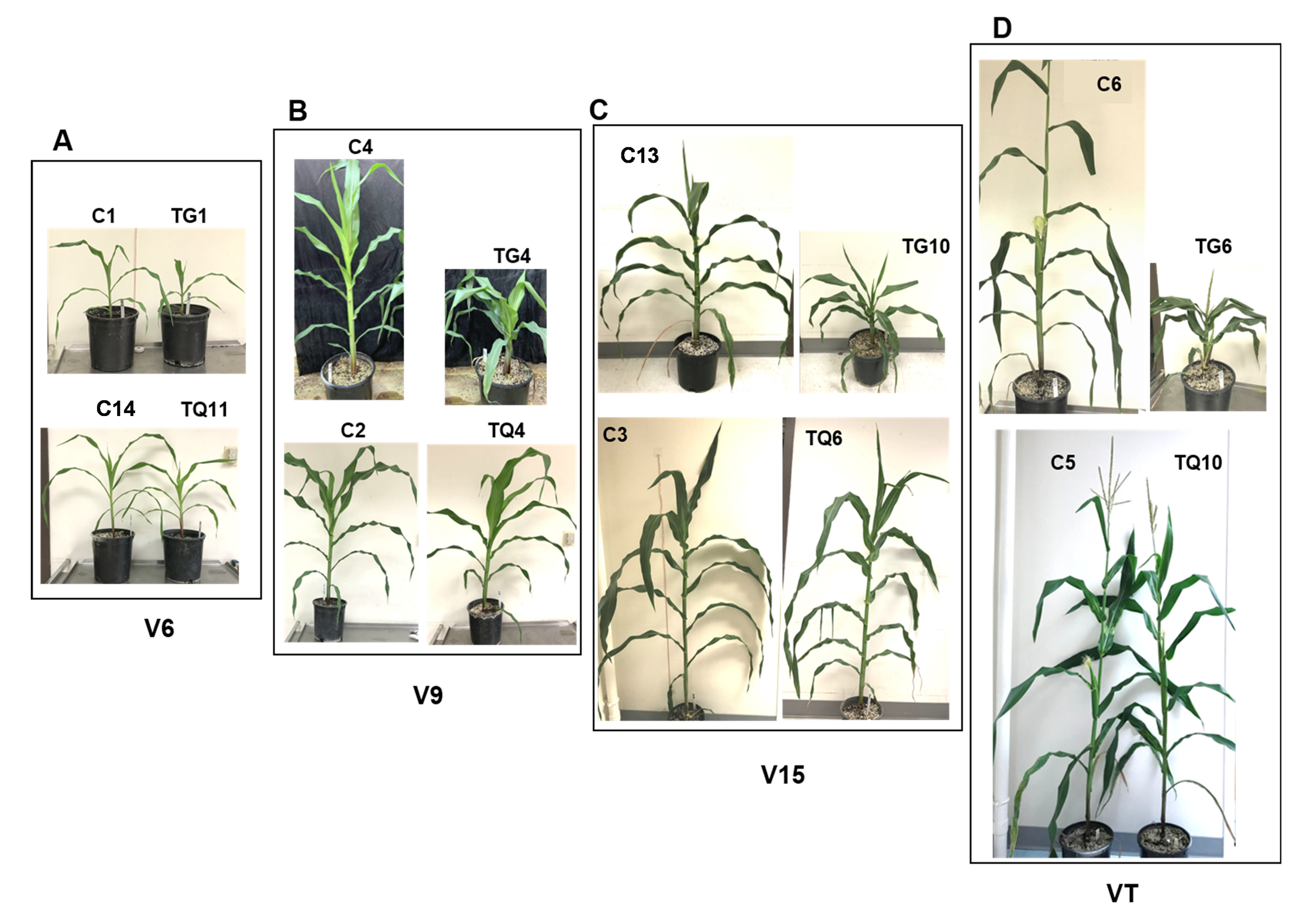

Supplement: S1 Fig — Plants TG1+C1 and TQ11+C10 at the V6 stage (A), TG4+C4 and TQ4+C2 at the V9 stage (B), TG10+C10 and TQ6+C3 at the V15 stage (C) and TG6+C6 and TQ10+C5 at the VT stage of development (D). (TIF) [file pone.0240369.s001.tif]

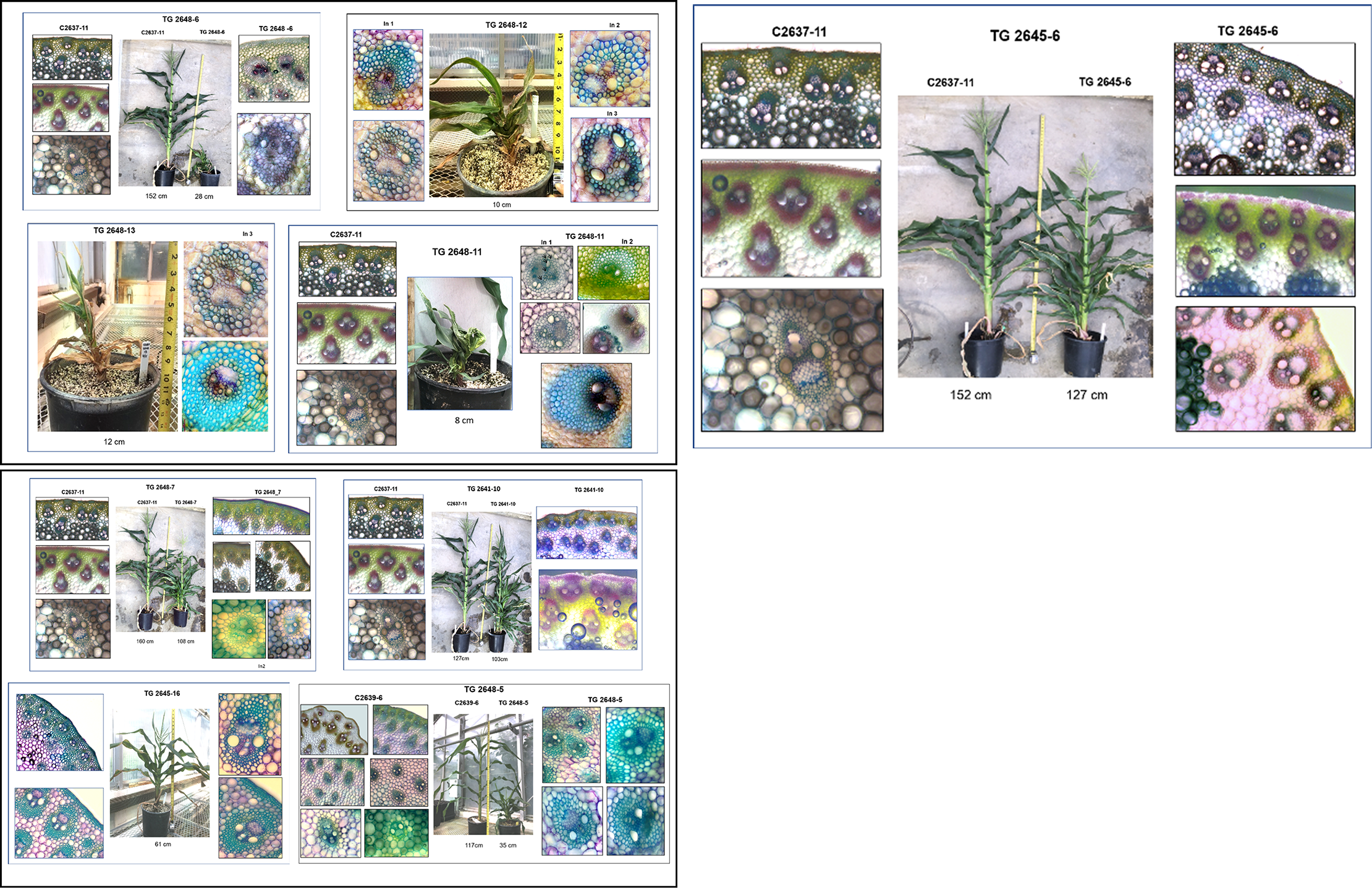

Supplement: S2 Fig — Nine plants from 127cm to 8cm high at maturity, and internode sections stained with phloroglucinol or toluidine blue, showing the relationship between plant height and the extent of cellular disruption of the vascular tissues (A-C). (TIF) [file pone.0240369.s002.tif]

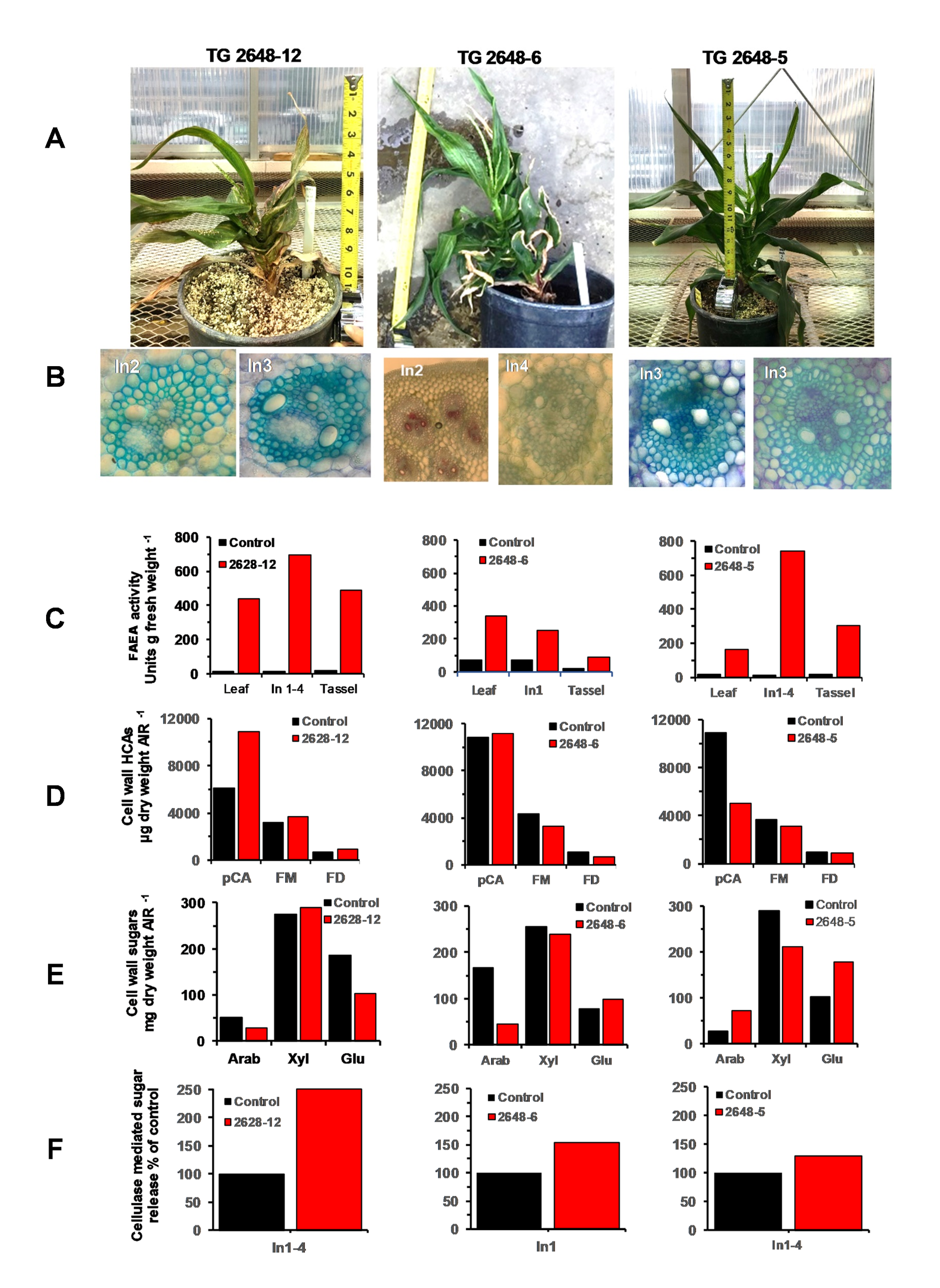

Supplement: S3 Fig — Plant phenotypes (A),transverse sections of internodes 2, 3, or 4 stained with either toluidine blue or phloroglucinol (B), FAEA activity of leaves internodes 1–4 and tassel (C), levels of cell wall HCAs of internodes 1–4; p-coumaric acid (pCA), ferulate monomers (FM) and ferulate dimers (FD) (D), cell wall sugars of internodes 1–4; arabinose (Arab), xylose (Xyl) and glucose (Glu) (E), and cellulase mediate release of total sugars from internodes 1–4 of the three mutant plants (F). (TIF) [file pone.0240369.s003.tif]

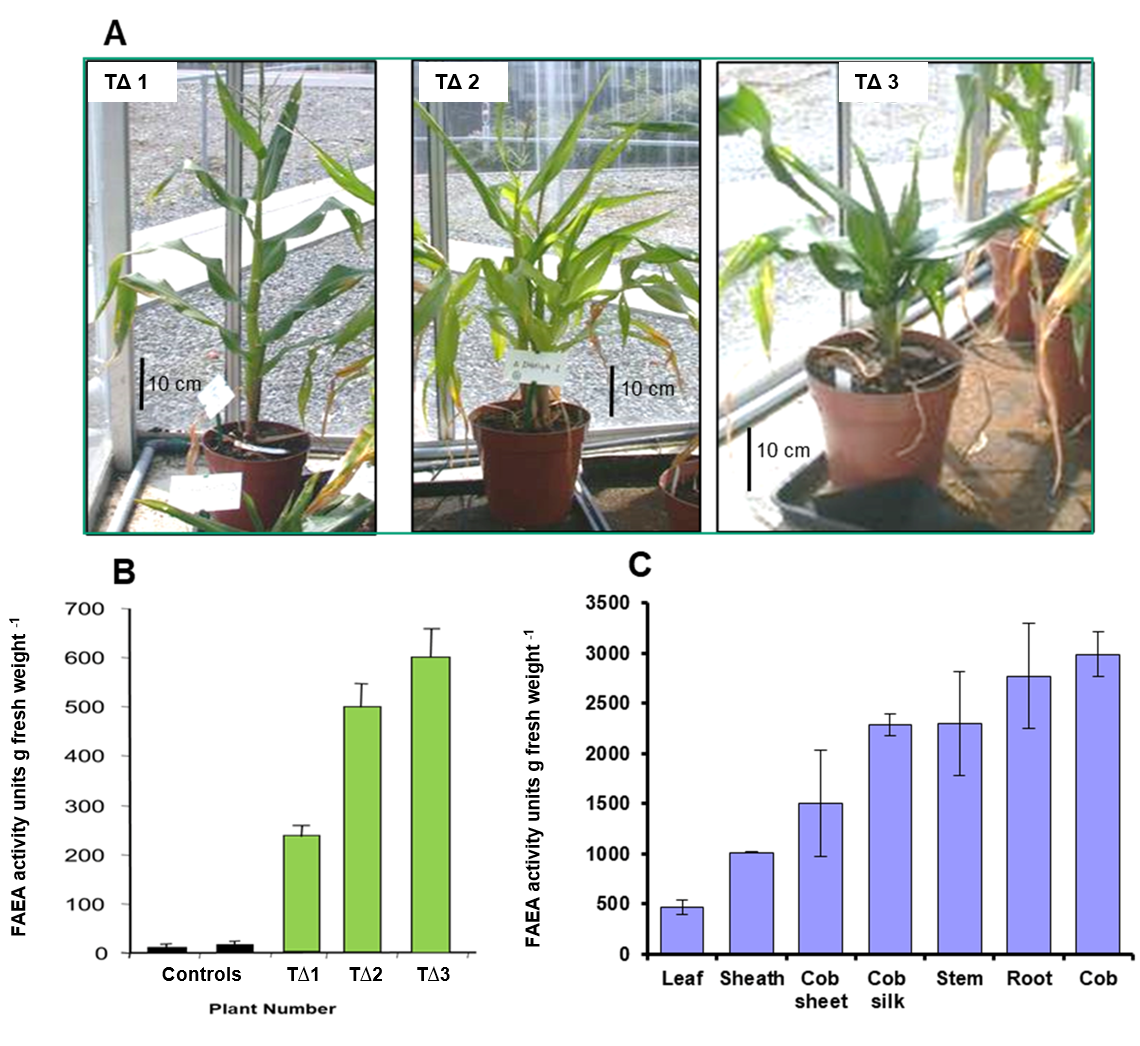

Supplement: S4 Fig — Plant phenotypes (A), FAEA activity in leaves compared with controls (B) and levels of FAEA activity of different tissues from a single plant TΔ4 (C). Tissues are ranked by FAEA activity. Mean ± seem (n = 3). (TIF) [file pone.0240369.s004.tif]

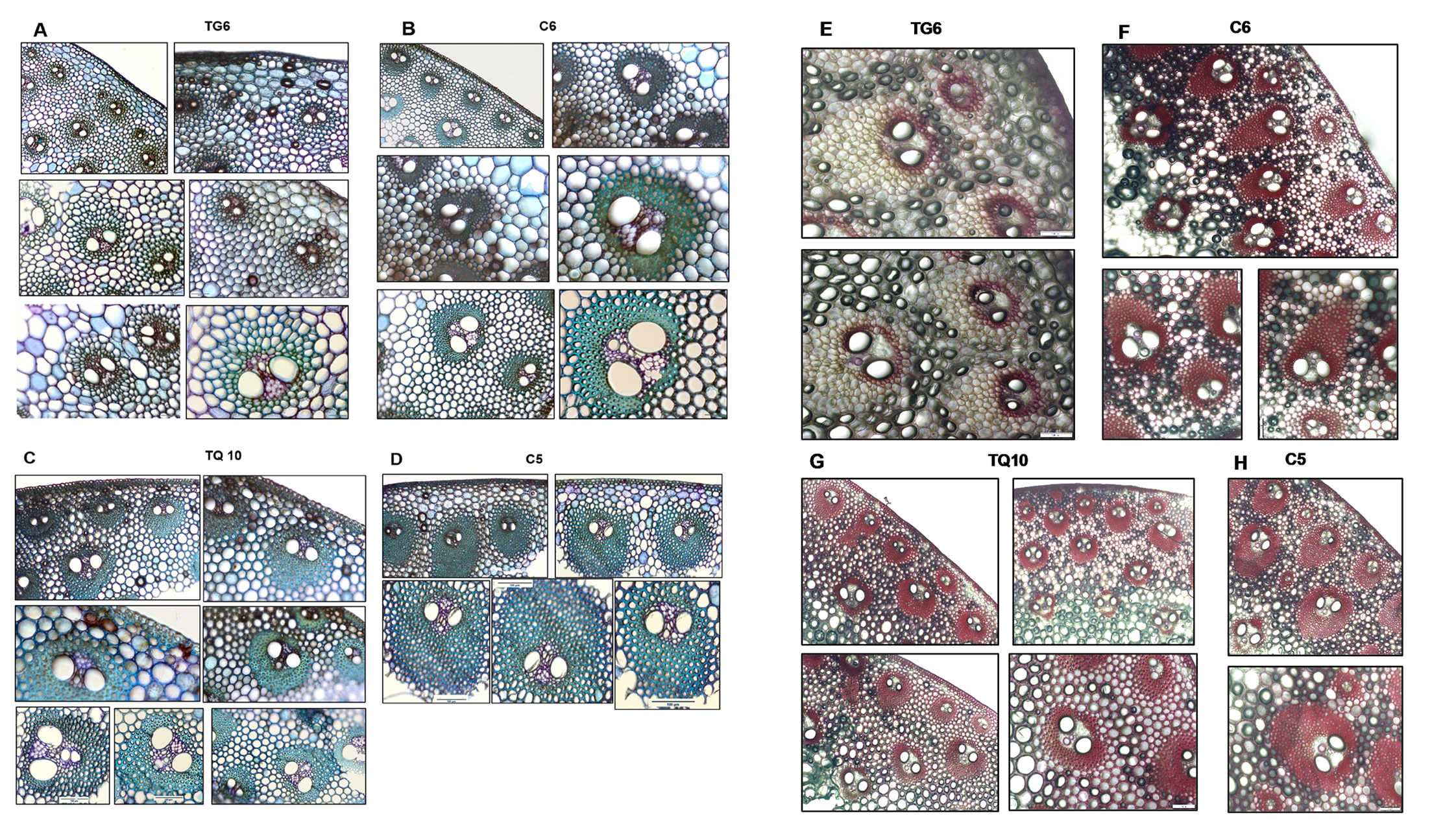

Supplement: S5 Fig — Transverse sections through the central vascular bundles of internode 6 of apoplast FAEA expressing plants. TG6 (A,E) and TQ10 (C,G), and control plants C6 (B,F) and C5 (D,H), grown under greenhouse conditions to the VT tasselling stage of development, stained with toluidine blue (A-D) or phloroglucinol (E-H). (TIF) [file pone.0240369.s005.tif]

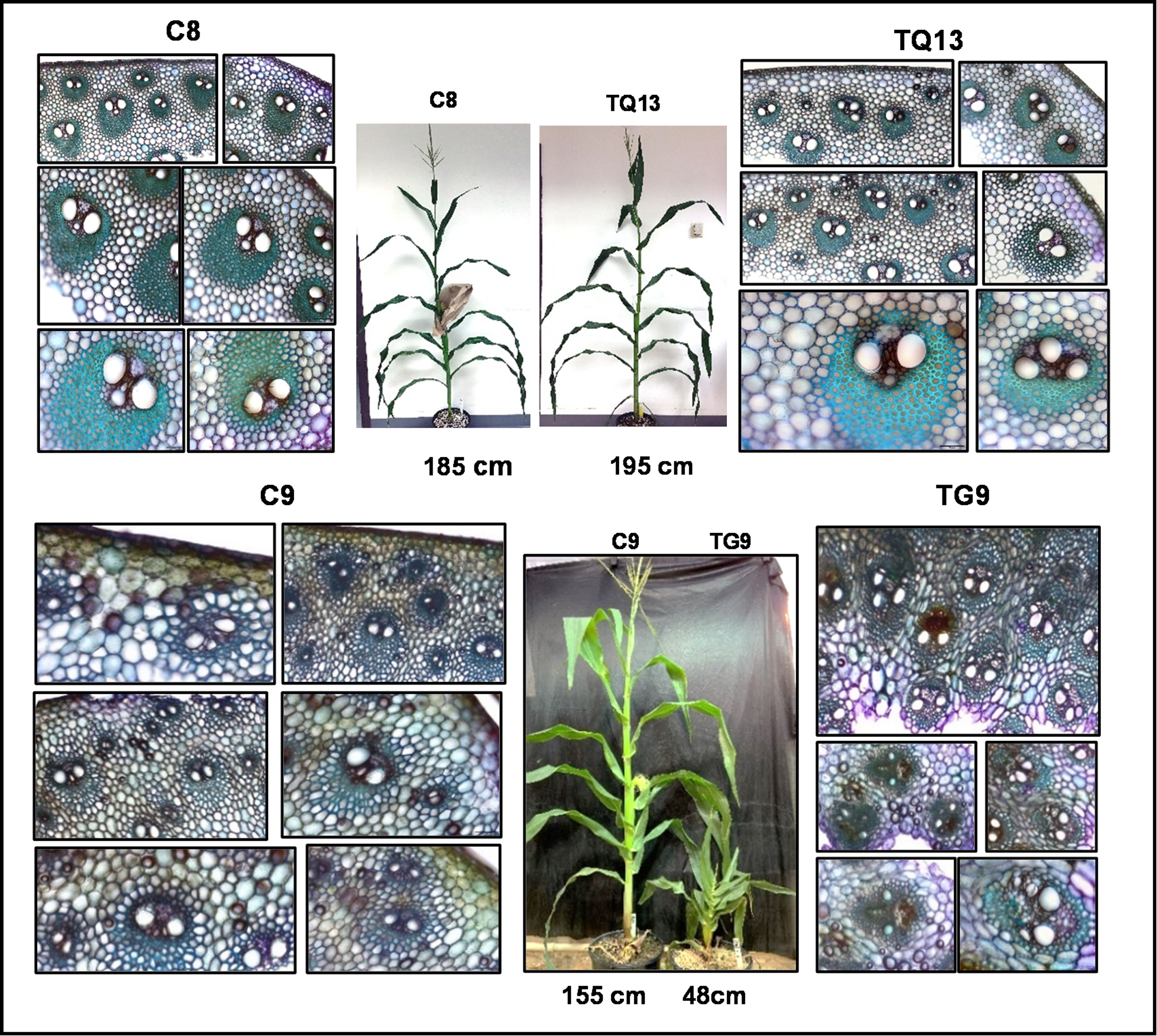

Supplement: S6 Fig — Transverse sections through the central vascular bundles of internode 6 of and phenotype of apoplast FAEA expressing plants TQ13 and TG9, and control plants C8 and C9, grown under greenhouse conditions to the VT tasselling stage of development, stained with toluidine blue. (TIF) [file pone.0240369.s006.tif]

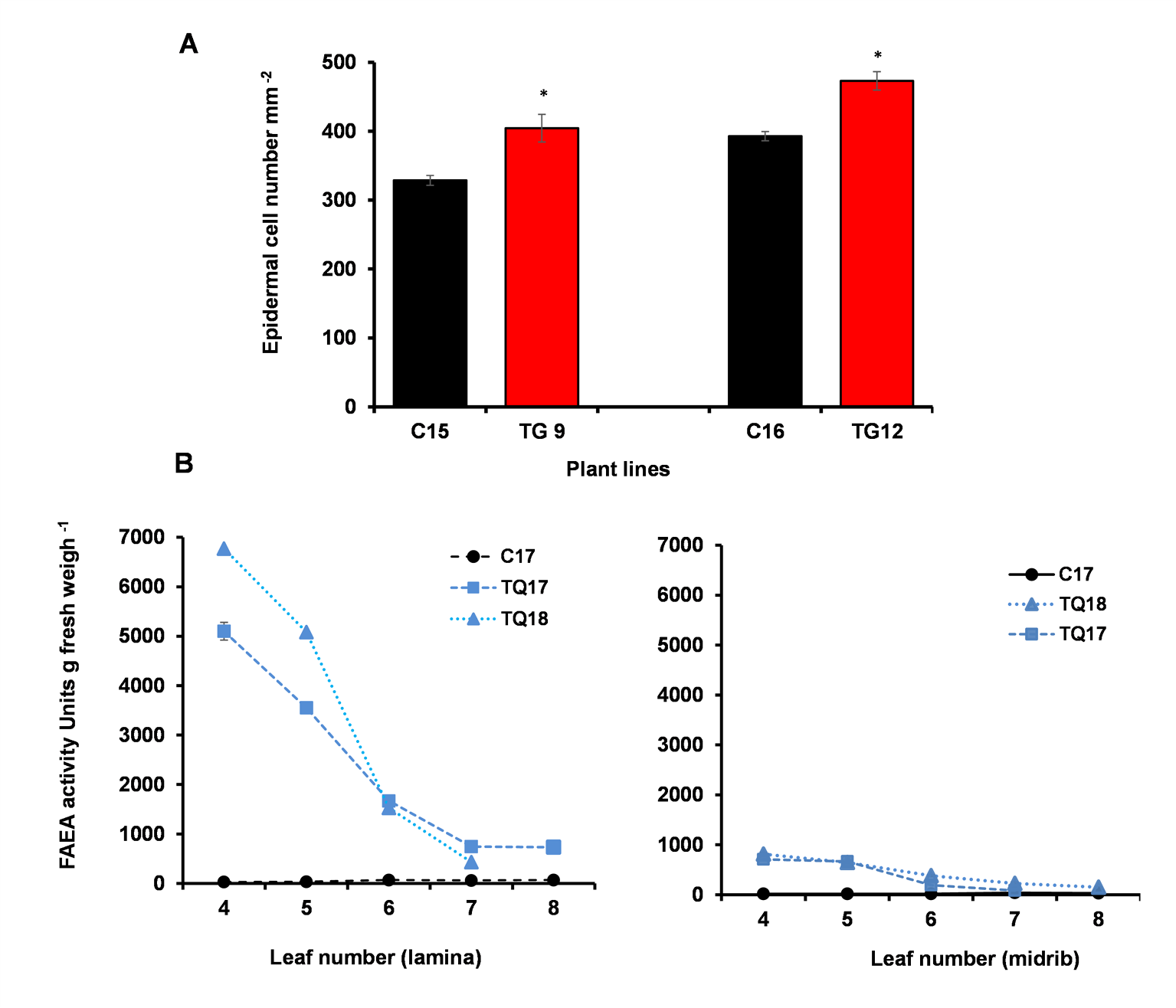

Supplement: S7 Fig — Number of epidermal cells mm -2 in terminal leaves of VT stage plants TG9 and TG12 compared with corresponding control plants C15 and C16. The number of cells was counted within 600,000 μ2 areas randomly selected from 10 leaf zones from 2 leaves per line (A). FAEA activity in leaf lamina and midribs of leaves 4 to 8 of V6 plants TQ17 and TQ 18 compared with a control plant C17 (B). Mean ± sem (n = 3). (TIF) [file pone.0240369.s007.tif]

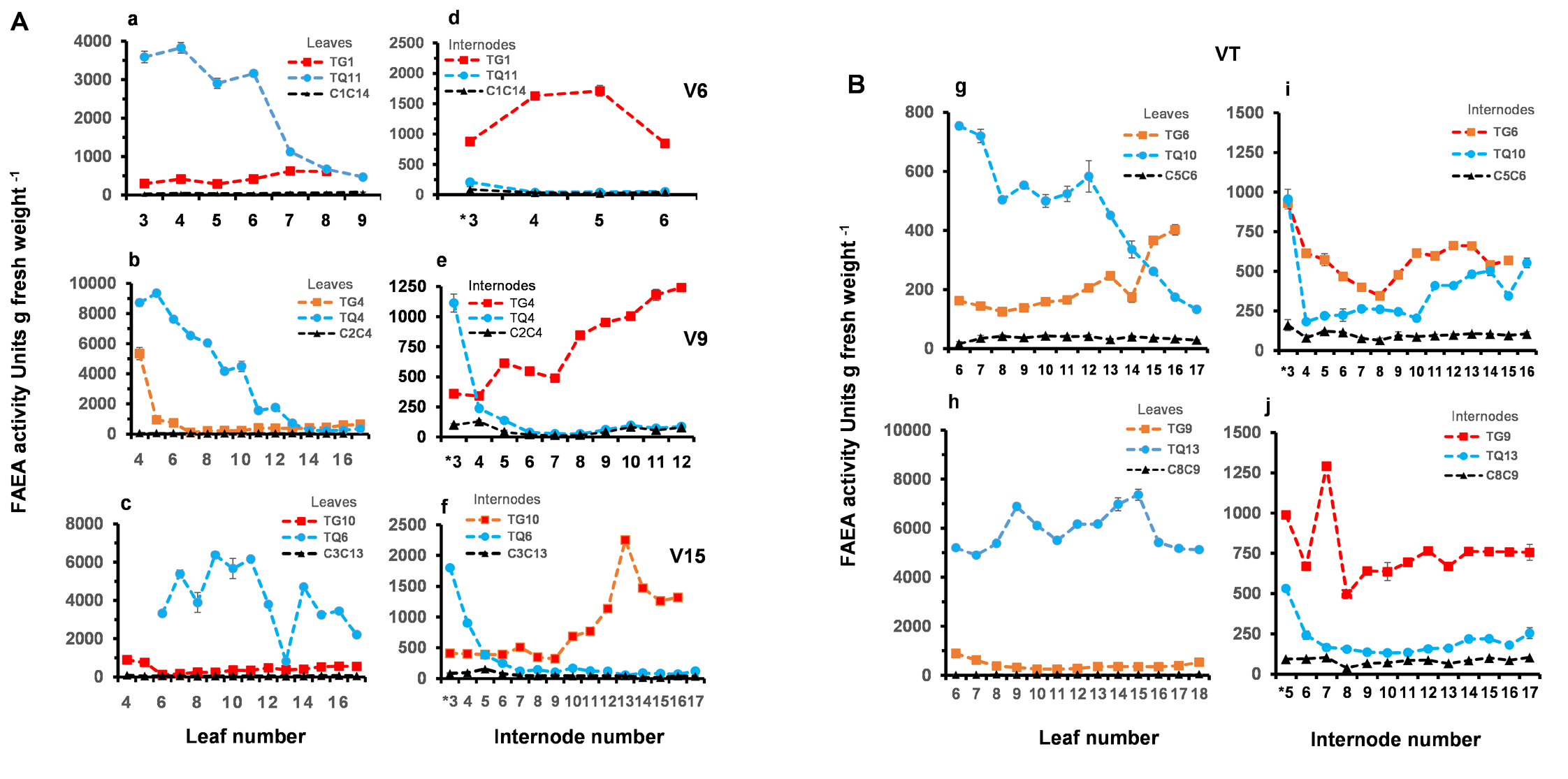

Supplement: S8 Fig — FAEA activity levels in (A) leaves (a-c) and internodes (d-f) at V6 (a,d), V9 (b,e), V15 (c,f), and (B) leaves (g-h) and internodes (i-j) at VT (g-j) developmental stages for TG1 and TQ11 (a,d), TG4 and TQ4 (b,e), TG10 and TQ6 (c,f),TG6 and TQ10 (g,i), TG9 and TQ13(h, j), and corresponding control plants (C1-C14, C2-C4,C3-C13, C5-C6 and C8-C9). Values for controls are the means ± sem (n = 6) of the TQ and TG control plants and values for TG ad TQ plants are the means ± sem (n = 3). * indicates the fused internodes 1–3. (TIF) [file pone.0240369.s008.tif]

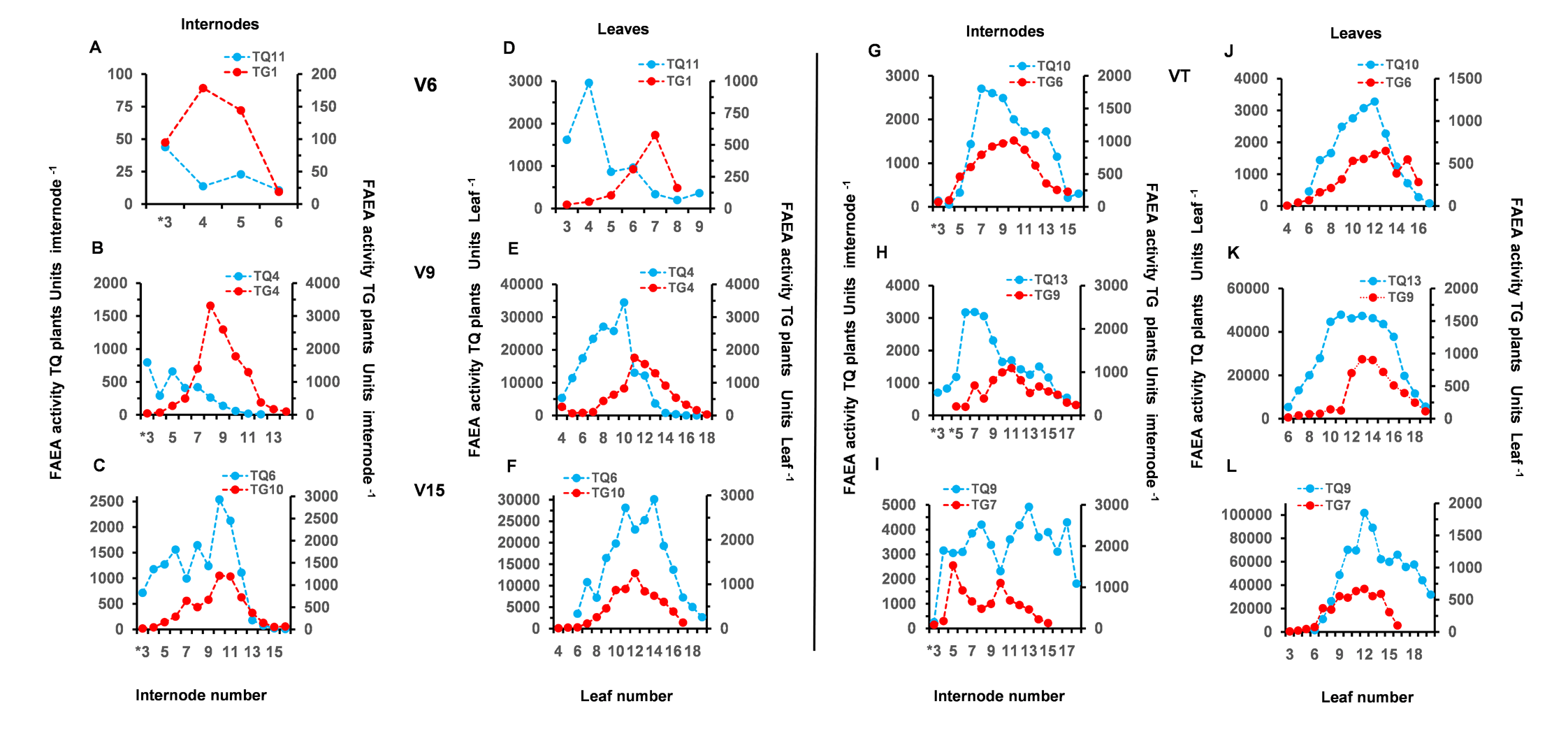

Supplement: S9 Fig — FAEA activity in internodes (A-C, G-I) and leaves (D-F, J-L) at V6 (A,D), V9 (B,E), V15 (C,F) and VT (G-L) developmental stages for TG1 + TQ11 (A,D), TG4 + TQ4 (B,E), TG10 + TQ6 (C,F) and for TG6+TQ10 (G,J), TG9,+ TQ13 (H,K), and TG7 + TQ9 (I-L). *indicates the fused internodes 1–3. (TIF) [file pone.0240369.s009.tif]

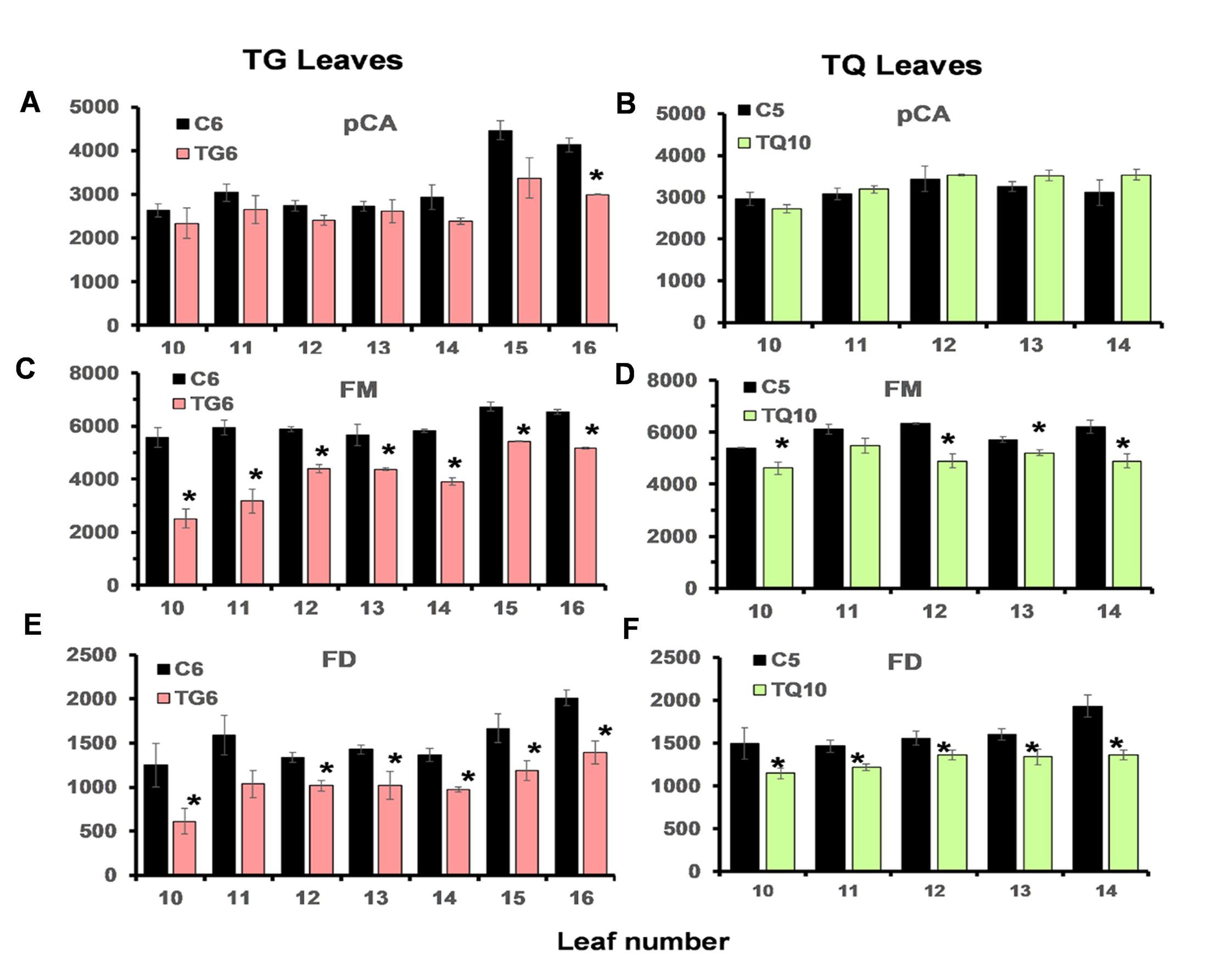

Supplement: S10 Fig — Levels of p-coumaric acid (A,B) ferulate monomers (C,D) and ferulate dimers (E,F) of selected leaves of plants TG6 + C6 control (A,C,E) and TQ10 + C5 control (B,D,F). Single determinations or means ± sem (n = 3). * indicate significant differences from controls (Student’s α = 0.05). (TIF) [file pone.0240369.s010.tif]
